# Beyond the cerebral cortex: cerebellar language-related subregions contributions to fluency in post-stroke aphasia

**DOI:** 10.1016/j.nicl.2026.103999

**Published:** 2026-04-29

**Authors:** Yuqian Zhan, Xiaohui Xie, Qiufang Ren, Xiaomin Pan, Zhishun Gao, Jin Li, Kai Wang, Tongjian Bai, Panpan Hu

**Affiliations:** a Department of Neurology, The First Affiliated Hospital of Anhui Medical University, Anhui Medical University, Hefei 230032, China; b Department of Neurology, The Second Affiliated Hospital of Anhui Medical University, Anhui Medical University, Hefei 230601, China; c Anhui Institute of Translational Medicine, Hefei 230032, China; d Institute of Artificial Intelligence, Hefei Comprehensive National Science Center, Hefei 230032, China; e Anhui Province Key Laboratory of Cognition and Neuropsychiatric Disorders, Hefei 230032, China

**Keywords:** Cerebellum, Fluency, Post-stroke aphasia, Functional connectivity

## Abstract

Although the classical language cortex significantly contributes to post-stroke aphasia (PSA), non-language-specific cortex, such as the cerebellum, is increasingly implicated in language. However, the specific contributions of its subregions to PSA, particularly regarding distinct language dimensions, remain unclear. Given fluency as a core dimension, we investigated the functional and structural integrity of cerebellar language-related subregions to clarify their distinct roles in fluent (FA) versus non-fluent aphasia (nonFA). We enrolled a primary cohort of 81 PSA patients (46 nonFA, 35 FA), and 77 healthy controls (HCs), alongside an independent external validation cohort (Aphasia Recovery Cohort [ARC]; 23 nonFA, 22 FA). Using individualized functional connectivity (FC) and volumetric analyses based on the Multi-Domain Task Battery (MDTB) atlas, we found that nonFA patients exhibited significantly decreased FC between the classical language network (LN) and language-related cerebellar subregions (right MDTB 8 and 9; R_MDTB8/9-LN FC), alongside reduced right Crus II volume. Correlation analysis revealed that these neuroimaging indicators were positively associated with language scores in nonFA, while no such relationships were observed in FA. Furthermore, mediation analysis indicated that right Crus II volume statistically accounted for the observed association between R_MDTB8/9-LN FC and overall Aphasia Quotient (AQ). As the key findings were replicated in the ARC, our results provide compelling evidence that the functional connectivity strength and structural integrity of specific cerebellar subregions contribute to language fluency. Our findings support expanding models of PSA beyond cortical regions and suggest that cerebellar-targeted strategies may improve language rehabilitation outcomes.

## Highlights

• Specific cerebellar subregions’ structure and function contribute to language.
• Non-fluent aphasia is linked to cerebellar-language network disruptions.
• Cerebellar volume statistically accounts for the functional-behavior link in non-fluent aphasia.
• The cerebellum is a validated, potential target for aphasia rehabilitation.

## Graphical abstract

**Figure:**
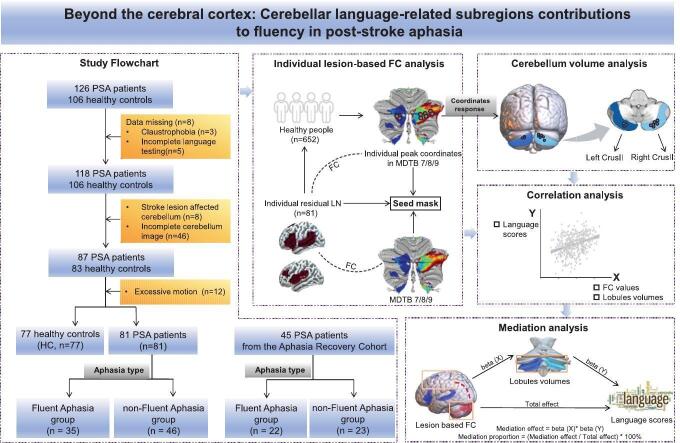

## 1 Introduction

Aphasia is one of the most devastating sequelae of stroke, with approximately 33% of survivors enduring persistent language deficits into the chronic phase (Keser et al., 2020). Post-stroke aphasia (PSA) can be classified by fluency, a core dimension of language, into fluent (FA) and non-fluent (nonFA) subtypes (Alyahya et al., 2020). Characterized by impaired fluency and laborious expression, nonFA exhibits more profound communication difficulties and a slower, more challenging recovery trajectory (Brady et al., 2025, You et al., 2023). The significant burden imposed on both individuals and society underscores the urgent need for a deeper understanding of the underlying neural mechanisms to develop targeted interventions (Kristinsson et al., 2022).

The perspective on the neural mechanisms of PSA is evolving from localized cortical lesions (Parvizi, 2009, Tremblay and Dick, 2016) to a model emphasizing network-level dysfunction (Jiang et al., 2025). Neuroimaging studies have identified a left-lateralized language network (LN), comprising classical language areas within the frontal and temporal cortices (Nair et al., 2015). Additionally, non-language-specific cortex, such as domain-general network (DGN), encompassing supplementary motor areas (SMA) and dorsal anterior cingulate cortices, also significantly contribute to language function (Turker et al., 2023, Malik-Moraleda et al., 2022). However, the majority of investigations have predominantly concentrated on cerebral cortical regions. Accumulating evidence has confirmed the potential role of the cerebellum in language processing (Strick et al., 2009, Lebel et al., 2021), particularly regarding output components of language (Ackermann et al., 2007).

The concept of a “linguistic cerebellum” is currently drawing attention. Associative diffusion MRI analyses revealed that there are contralateral interconnections between the cerebellum and the cerebral cortex via the thalamus, and the dentate nucleus is structurally connected to various areas in the contralateral frontal lobe (Ji et al., 2019). Functional neuroimaging studies in healthy individuals have demonstrated significant activation of the right cerebellum during verb generation tasks (Price, 2012), and inhibitory stimulation of the right cerebellum can reduce verbal output (Arasanz et al., 2012). Furthermore, damage on cerebellum has been shown to affect verbal fluency (Satoer et al., 2024). Research on PSA has further investigated the alterations within specific anatomical subregions. For instance, Zhang et al. found that decreased functional connectivity (FC) between the right Crus I and left precentral gyrus, alongside reduced gray matter volumes (GMV) in right lobule VI and Crus I in PSA (Zhang et al., 2022). Another study demonstrated reduced fractional amplitude of low-frequency fluctuations (fALFF) in right cerebellar Crus I (Cheng et al., 2023). Although these findings highlight the involvement of cerebellar subregions, the precise contribution of these areas to specific dimensions of language, remains unclear. While recent work by Stilling et al. (Stilling et al., 2025) compellingly demonstrates that Crus I/II connectivity with cortical language regions significantly predicts naming outcomes in chronic aphasia, their characterization of cerebellar contributions primarily adheres to traditional anatomical boundaries.

Cerebellar functional networks are not strictly constrained by lobular anatomy (King et al., 2019). To address this complexity, King et al. has proposed a Multi-Domain Task Battery (MDTB) to map the cerebellum into 10 discrete regions with specific functions (King et al., 2019). Specifically, they found that regions MDTB7 and MDTB8 (largely within bilateral Crus I/II) primarily correlated with narrative and word comprehension respectively, while region MDTB9 (right-hemispheric, lateral to region MDTB8) was mainly involved in verbal fluency. Building on this foundational work and considering the link between the cerebellum and fluency, the contributions of these functionally-defined subregions to this core language dimension in PSA warrants investigation. Moreover, given the established influence of cerebellar morphology on language (Zhang et al., 2022, Newman-Norlund et al., 2024), its specific role in the relationship between FC and language performance deserves further examination.

Therefore, we aimed to elucidate the role of functionally-defined cerebellar subregions in fluency by investigating functional and structural differences between nonFA and FA, and relating these neural measures to language performance. We hypothesize that individuals with nonFA would exhibit a distinct pattern of functional disconnection and structural atrophy within specific cerebellar subregions, with these alterations being significantly associated with language deficits, differing from that of FA. Furthermore, we posited that structural integrity would mediate the influence of functional connectivity on language performance. Elucidating these specific cerebellar subregions as critical contributors may establish them as novel targets for therapeutic interventions, aimed at improving language outcomes in individuals with aphasia.

## 2 Methods

### 2.1 Study design and participants

**Fig. 1:**
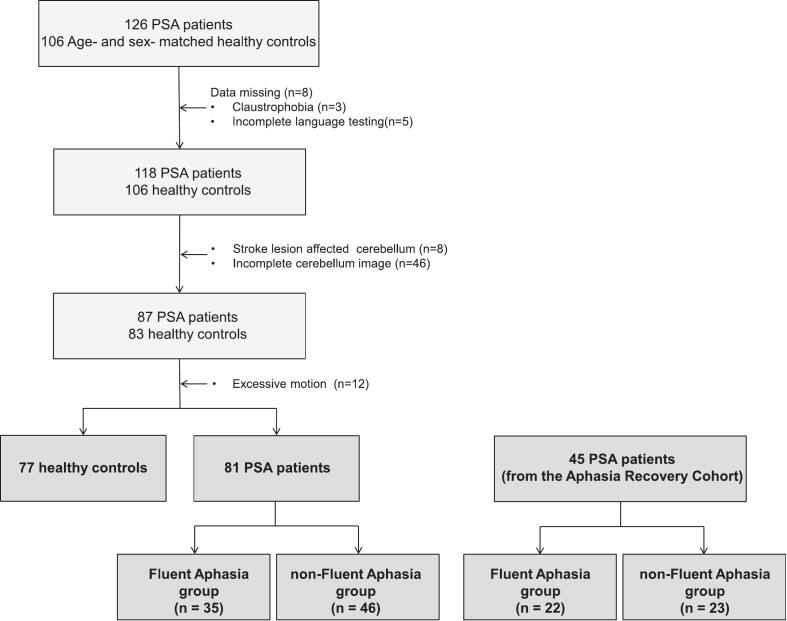
Flowchart depicting the participant inclusion process. Abbreviation: PSA = post-stroke aphasia; FA = fluent aphasia; nonFA = non-fluent aphasia.

To establish reliable and generalizable findings, this retrospective, cross-sectional study included a primary cohort and an independent external validation cohort (Fig. 1).

The primary cohort included PSA patients from the First Affiliated Hospital of Anhui Medical University and Anhui Provincial Hospital of Acupuncture and Moxibustion in Hefei, China. The initial cohort comprised 126 patients with first-ever, unilateral left-hemisphere stroke-induced aphasia, alongside 106 HCs matched for key demographic variables, including age, sex, education level. Inclusion criteria were: (i) first-ever ischemic or hemorrhagic stroke confined to the left hemisphere resulting in aphasia; (ii) right-handed; (iii) an age range of 25 to 80 years; (iv) native Chinese speaker. Exclusion criteria, designed to minimize confounding factors, were: (i) cerebellar or right-hemisphere involvement; (ii) neurological disease or family of hereditary mental disorder; (iii) severe dysarthria; (iv) head injury or surgery history (v) alcohol or substance abuse; (vi) cerebral tumor or abscess; (vii) claustrophobia or implants incompatible with MRI; (viii) excessive motion (> 3 mm in translation or > 3◦ in rotation) during scanning and abnormal brain structure. After screening, 45 patients were excluded, resulting in a final cohort of 81 PSA patients and 77 HCs, and PSA was further classified into nonFA (n = 46) and FA (n = 35) subgroups (Table 1).

**Table 1:** Demographic and clinical characteristics of the HC and PSA patients in primary cohort.

group	HC (n = 77)	PSA (n = 81)		T-test		ANOVA
		FA (n = 35)	nonFA (n = 46)	*p* value (HC and PSA)	*p* value (FA and nonFA)	*p* value
Age (years)	55.70 ± 11.44	51.66 ± 11.67	59.48 ± 9.77	0.676 ^d^	0.002 ^c^	0.008 ^a^
Sex (male/female)	50/27	24/11	31/15	0.693 ^b^	0.910 ^b^	0.919 ^b^
Education (years)	9.38 ± 3.76	9.77 ± 5.18	7.46 ± 4.14	0.229 ^d^	0.017 ^d^	0.022 ^a^
Total intracranial volume (cm^3^)	1520.85 ± 125.04	1547.66 ± 114.43	1518.39 ± 128.18	0.711 ^d^	0.256^c^	0.503 ^a^
Disease duration (weeks)	−	28.00 ± 78.88	8.57 ± 13.53	−	0.549 ^d^	−
Lesion volume (cm^3^)	−	39.38 ± 39.36	50.08 ± 53.03	−	0.375 ^d^	−
Preserved LN volume (cm^3^)	−	23.85 ± 3.47	23.19 ± 5.09	−	0.882 ^d^	−

Language scores						
Fluency ^e^	−	7.26 ± 1.65	1.78 ± 1.84	−	<0.001 ^d^	−
AQ scores ^e^	−	66.45 ± 22.19	26.74 ± 21.98	−	<0.001 ^d^	−

Note: ANOVA = analysis of variance; HC = healthy controls; PSA = post stroke aphasia; FA = fluent aphasia; nonFA = non-fluent aphasia; AQ = aphasia quotient. Bold indicates statistical significance (*p* < 0.05). Continuous variables are presented as the means ± SD, and categorical variables are presented as counts (n). ^a^ ANOVA between HC, FA and nonFA groups. ^b^ Chi-squared test. ^c^ independent samples *T*-test between FA and nonFA. ^d^ Mann-Whitney *U* test. ^e^ standardized scores according to Western Aphasia Battery.

To validate our findings, we examined a cohort of 45 patients from the publicly available Aphasia Recovery Cohort (ARC) database (Gibson et al., 2024) (https://doi.org/10.18112/openneuro.ds004884.v1.0.1). This validation subset was then stratified into nonFA (n = 23) and FA (n = 22) subgroups (Table 2).

**Table 2:** Demographic and clinical characteristics of PSA patients in ARC.

group	PSA (n = 45)		*p* value
	FA (n = 22)	nonFA (n = 23)	
Age (years)	62.64 ± 11.85	58.91 ± 12.71	0.324 ^a^
Sex (male/female)	12/10	12/11	0.873 ^b^
Disease duration (weeks)	114.4 ± 104.6	217.3 ± 303.2	0.665 ^a^
Lesion volume (cm^3^)	55.32 ± 52.41	115.7 ± 68.42	<0.001 ^a^
Language scores			
AQ scores ^d^	82.32 ± 11.51	41.18 ± 15.38	<0.001 ^c^

Note: PSA = post stroke aphasia; FA = fluent aphasia; nonFA = non-fluent aphasia; AQ = aphasia quotient. Bold font for *p*-values indicates statistical significance (*p* < 0.05). Continuous variables are presented as the means ± SD, and categorical variables are presented as counts (n). ^a^ Mann-Whitney *U* test. ^b^ Chi-squared test. ^c^ independent samples *T*-test. ^d^ standardized scores according to Western Aphasia Battery.

The research adhered to the Declaration of Helsinki and was approved by the Anhui Medical University Ethics Committee (2019H009). Written informed consent was obtained from all participants or their legal guardians. The Institutional Review Board (IRB) at the University of South Carolina deemed the anonymized ARC data, with variables generalized to comply with safe harbor criteria, exempt from further review (proposal ‘OpenNeuro Clinical’s Pro00132576).

### 2.2 Language assessment

In the primary cohort, a certified speech-language pathologist conducted comprehensive language profiling at baseline (prior to MRI) using the Western Aphasia Battery-revised (WAB-R) (Kertesz, 1982, Kertesz, 2007), and Aphasia Battery of Chinese (ABC) (Surong, 1992), a validated and culturally adapted version of WAB. The ABC protocol yields quantitative scores across four key linguistic dimensions: spontaneous speech (max score: 20), auditory comprehension (max score: 230), repetition (max score: 100), and naming (max score: 80). Aphasia quotient (AQ) was derived through weighted composite scoring of domain-specific performance metrics, with scores below 93.8 constituting clinical diagnosis of aphasia according to validated criteria (Zhang et al., 2018). To categorize patients into FA and nonFA subgroups, we strictly applied the foundational taxonomic criteria of the WAB framework (Kertesz and Phipps, 1977, Kertesz, 1982). Within the spontaneous speech evaluation, which comprises a 10-point information scale and a 10-point fluency scale, the specific fluency score served as the definitive diagnostic determinant. Utilizing the established operational cut-off, patients demonstrating a fluency score of ≤ 4 were classified into the nonFA group, whereas those achieving a score of ≥ 5 were categorized into the FA group.

In the independent ARC validation cohort, AQ scores and categorical aphasia classifications based on the standard WAB protocol, were obtained from an open-source repository. Because the database did not provide specific fluency scores for individual patients, we assigned patients to the FA or nonFA groups based directly on their provided WAB subtypes.

### 2.3 Neuroimaging

#### 2.3.1 MRI data acquisition

The MRI data of the primary cohort were acquired on a 3.0 T GE Discovery 750 scanner (GE Healthcare, Milwaukee, WI, USA) at the University of Science and Technology of China. High-resolution T1-weighted anatomical images were acquired using a 3D BRAVO sequence with the following parameters: repetition time (TR)/echo time (TE) = 8.16/3.18 ms; inversion time = 450 ms; flip angle = 12°; field of view (FOV) = 256 × 256 mm^2^; matrix = 256 × 256; slice thickness = 1 mm (no gap); 188 sagittal slices; voxel size = 1 × 1 × 1 mm^3^. Resting-state functional MRI (rsfMRI) were acquired with a gradient-echo echo-planar imaging (EPI) sequence: TR/TE = 2400/30 ms; flip angle = 90°; FOV = 192 × 192 mm^2^; matrix = 64 × 64; slice thickness = 3 mm (no gap); and 46 transverse slices parallel to the anterior-posterior commissure (AC-PC) line, 217 volumes.

MRI data of ARC were acquired on a Siemens Trio 3.0 T scanner at the University of South Carolina. T1-weighted images were acquired using an MP-RAGE sequence with 1  mm isotropic voxels, a 256 × 256 matrix size, 9° flip angle, and 192 slices (TR = 2250 msec, TI = 925 msec, TE = 4.15) with parallel imaging (GRAPPA = 2, 80 reference lines). Rs-fMRI: Multiband sequence (x2) with a 216 × 216  mm field of view, a 90 × 90 matrix size, and a 72° flip angle, 50 axial slices (2  mm thick with 20% gap yielding 2.4  mm between slice centers), TR = 1650 msec, TE = 35 msec, GRAPPA = 2, 44 reference lines, interleaved ascending slice order, 427 volumes (Yourganov et al., 2021).

Throughout the functional scan, participants were instructed to remain awake with their eyes closed and minimize head movement. A detailed comparison of the MRI acquisition parameters, preprocessing pipelines, and other relevant characteristics for both the primary and validation cohorts is provided in Supplementary Table S1.

#### 2.3.2 Lesion Delineation and normalization

In the primary cohort, stroke lesions were manually delineated on each patient’s native-space T1-weighted anatomical image using MRIcron software. To ensure anatomical fidelity, a board-certified neurologist independently reviewed and confirmed each segmentation. In the ARC, lesion masks were created based on delineations of chronic infarcts on T2-weighted images.

**Fig. 2:**
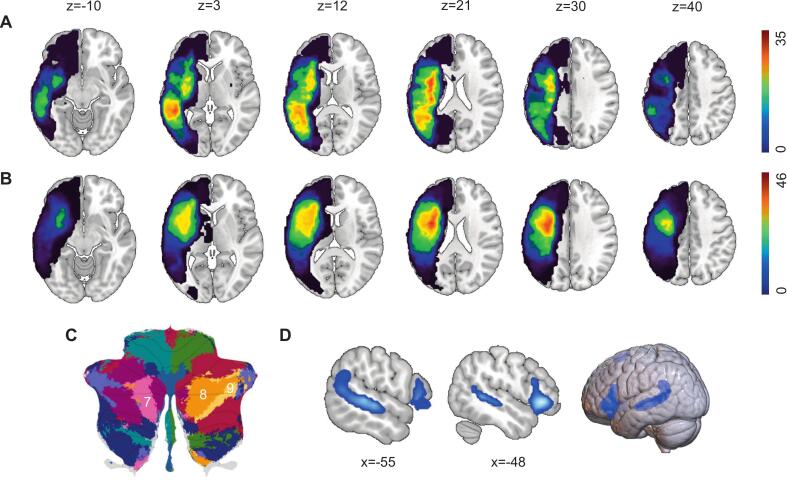
Lesion density overlap map among aphasia patients. (A) Lesion-overlap heat-map for the FA group (n = 35); warmer colors indicate a higher proportion of subjects with damage at a given voxel; Color bar indicates the number of patients; X and Z refer to the x-plane and z-plane coordinates of the MNI space. (B) Lesion-overlap heat-map for the nonFA group (n = 46) displayed using the identical threshold and color scale as in A. (C) Localization of cerebellar functional regions MDTB7 (pink), MDTB8 (orange), and MDTB9 (yellow) on the Multi-Domain Task Battery atlas. (D) Localization of LN. Abbreviation: FA = fluent aphasia; nonFA = non-fluent aphasia; MDTB = Multi-Domain Task Battery; LN = classical language network. (For interpretation of the references to colour in this figure legend, the reader is referred to the web version of this article.)

These binary lesion masks were spatially normalized to the Montreal Neurological Institute (MNI152) standard space by applying transformation parameters obtained from the registering each patient’s T1-weighted image to the MNI152 template. Finally, to visualize the anatomical distribution of damage, group-level lesion overlap maps were constructed via a voxel-wise summation of the normalized masks for FA and nonFA subgroups (Fig. 2A-B).

#### 2.3.3 Defining the cortical language network and cerebellar regions of interest

The LN was defined utilizing a *meta*-analytically derived mask from the Neurosynth database (search term: “language”; https://neurosynth.org), encompassing canonical language areas. To ensure that subsequent connectivity analyses accurately reflected individual lesion patterns, we first constructed a unique “preserved LN” for each patient by excluding lesioned voxels from the standard LN mask. This individualized approach could minimize the confounding effects of lesion-induced alterations across patients.

Cerebellar regions of interest (ROIs) were delineated through a multi-step, hypothesis-driven procedure. First, we utilized the established MDTB functional atlas to identify language-related cerebellar areas (MDTB 7, 8, and 9; Fig. 2C) (King et al., 2019). Next, we computed voxel-wise FC maps between each patient’s preserved LN and each of the predefined cerebellar regions. To address lesion heterogeneity and potential individualized functional reorganization, we employed a novel data-driven approach to identify patient-specific connectivity peaks. These peaks were derived exclusively from an independent normative functional connectivity dataset of 652 healthy participants (Ji et al., 2023), entirely independent of the patients’ own fMRI data. Specifically, each patient’s unique “preserved LN” was utilized as a seed, which was then mapped onto this independent normative dataset to precisely identify the peak connectivity coordinate for that individual. This methodology, conceptually aligned with established lesion network mapping frameworks (Fox, 2018, Boes et al., 2015) and similar to recent network localization studies (Corp et al., 2019, Joutsa et al., 2022), ensures statistical independence and leverages the high signal-to-noise ratio inherent in a large healthy cohort (Cash et al., 2020). Within each cerebellar subregion, we determined the individual peak FC coordinate for each patient. Remarkably, the peak FC coordinate consistently localized to the Crus II lobule of the right cerebellum across three MDTB subregions (R_MDTB7, R_MDTB8, and R_MDTB9). To assess hemispheric laterality, we then identified the corresponding homologous coordinates in the left MDTB 7 and 8 (L_MDTB7, L_MDTB8; MDTB9 lacks a left cerebellar homologue) to serve as control regions. For every identified coordinate (individual peak locus and their contralateral controls), a spherical ROI with a 6-mm radius was created using the MarsBar toolbox, a size chosen to optimize spatial specificity and signal-to-noise ratio (Marrelec and Fransson, 2011). The MNI coordinates for each ROI, which demonstrated high consistency across patients, are provided in Supplementary Table S2.

To formally quantify the benefit of this individualized approach, we conducted a direct comparison against a standard atlas-based centroids method. For this, we also computed functional connectivity from the atlas-defined centroid of each cerebellar ROI to the patient’s preserved language network. We then employed hierarchical regression models to contrast the predictive power of the atlas-based FC versus our individualized peak FC in explaining aphasia severity, and further compared the model fit using the Akaike Information Criterion (AIC).

#### 2.3.4 Functional MRI preprocessing and FC analysis

Functional images were preprocessed using the Data Processing Assistant for Resting-State fMRI toolkit (Chao-Gan and Yu-Feng, 2010) (DPARSF: https://rfmri.org/DPARSF) and the Statistical Parametric Mapping 12 (SPM12: https://www.fil.ion.ucl.ac.uk/spm/software/spm12/) in MATLAB (R2023b). The first ten volumes of each scan were discarded to allow for T1 signal stabilization, resulting in 207 usable time points (approximately 497 s) for the primary cohort and 417 usable time points (approximately 688 s) for the validation cohort. The remaining volumes underwent slice-timing correction, realignment for head motion correction, and co-registration to the individual’s T1-weighted anatomical image. Anatomical images were then used to compute transformations for spatial normalization into MNI space via the Diffeomorphic Anatomical Registration Through Exponentiated Lie Algebra (DARTEL) algorithm. Following normalization, nuisance regression was performed to remove signal contributions from the global mean signal, 24 Friston motion parameters, as well as mean signals from white matter and cerebrospinal fluid. Finally, functional images were spatially smoothed using a 4-mm full-width at half-maximum (FWHM) Gaussian kernel and temporally band-pass filtered (0.01–0.1 Hz).

For lesion-based individualized resting-state FC analysis, evaluated using DPARSF software between seed regions, mean time series were extracted, and pairwise Pearson’s correlations were computed and Fisher’s *r*-to-*z* transformed to generate standardized *z*-score metrics of connectivity strength. To approximate the lesion distribution observed in the patient cohort while maintaining anatomical correspondence, each patient’s preserved language network was individually projected onto a single representative healthy brain. For each projection, corresponding time series were extracted, and FC maps computed; these 81 resultant maps were then averaged to define the HC’s FC baseline. This approach enhances comparability by aligning seed definition and signal extraction across groups and reduces variability related to lesion-induced differences in seed morphology. At the same time, this strategy entails a methodological tradeoff, as the resulting healthy reference is partially constrained by patient-specific lesion geometry and therefore does not represent a fully intact, conventional language network baseline.

#### 2.3.5 Structural MRI preprocessing and GMV analysis

Cerebellar GMV was analyzed with the spatially unbiased infratentorial toolbox (SUIT, version 3.7) in SPM12 (Diedrichsen et al., 2011). T1-weighted images underwent automated segmentation, generating grey matter, white matter, and cerebrospinal fluid probability maps, with simultaneous isolation of the cerebellum with SUIT routines. For more accurate alignment, individual cerebellums were nonlinearly registered to the high-resolution SUIT atlas template (1 mm^3^ isotropic) employing the DARTEL algorithm, yielding participant-specific deformation fields. These fields were subsequently applied to warp the native-space GM probability maps into the standard SUIT template space. Importantly, this spatial normalization process incorporated modulation with Jacobian determinants derived from the deformation fields, which preserved local GM volume information during warping. The quality of each cerebellar segmentation and normalization was visually confirmed by two raters blinded to group membership.

Our selection of the right Crus II for volumetric analysis was directly guided by our FC findings, where the peak coordinate consistently localized to the right Crus II. This led us to form the specific hypothesis that these functional alterations might have a structural correlate. To test this, we targeted the right Crus II for volumetric analysis and included the homologous contralateral region, the left Crus II, as an anatomical control. Given that the ‘modulation’ step in SUIT, which scales voxel-wise GM probability by the Jacobian determinants of the deformation fields, inherently corrects for global brain size differences relative to the template space, total intracranial volume (TIV) was not included as an explicit covariate in subsequent primary models, which is consistent with prior studies employing similar normalization and modulation approaches (Guo et al., 2016, O'callaghan et al., 2016, Diedrichsen et al., 2009). Nevertheless, to ensure the rigor and completeness of our methodology, we conducted an additional sensitivity analysis that explicitly controlled for estimated TIV, as detailed in Section 2.4.3. The estimated TIV was calculated using FreeSurfer version 7.4.1 using T1-weighted images in the software (Buckner et al., 2004).

### 2.4 Sensitivity analysis

#### 2.4.1 Controlling for demographic differences

To confirm our findings were robust against demographic differences, we performed a sensitivity analysis by replicating the primary ANCOVA models within a demographically matched subsample. This allowed for a direct assessment to ensure the observed effects were not driven by baseline group variations.

#### 2.4.2 Controlling for preserved LN volume

To confirm our findings were not confounded by the volume of the remaining LN, we conducted a sensitivity analysis. All key statistical models were replicated, including ANCOVAs, correlations, and mediation analyses, with preserved LN volume systematically replacing lesion volume as the covariate.

#### 2.4.3 Controlling for total intracranial volume

To further ensure that our observed group differences in Crus II volume were not confounded by variations in overall brain size, we conducted a sensitivity analysis by controlling for total intracranial volume (TIV). Although the modulation step in the SUIT toolbox preprocessing inherently accounts for individual differences in head and brain size (Guo et al., 2016, O'callaghan et al., 2016, Diedrichsen et al., 2009), we calculated each participant’s TIV using FreeSurfer and included it as an additional covariate in the ANCOVA models that examined group differences in Crus II volume.

#### 2.4.4 Controlling for head motion

Functional connectivity is highly susceptible to motion-induced noise. In the original preprocessing pipeline, we excluded subjects with excessive motion (> 3 mm in translation or > 3° in rotation). To confirm our findings were robust against the confounding effects of head motion, we performed a stricter sensitivity analysis. This involved limiting our sample to participants with a mean framewise displacement (FD) of less than 0.2 mm (Power et al., 2011, Van Dijk et al., 2011).

#### 2.4.5 Controlling for global signal regression

To confirm our findings were not dependent on the inclusion of global signal regression, we conducted a further sensitivity analysis. This involved replicating our key statistical models within the same low-motion subsample (mean FD < 0.2 mm), but systematically omitting global signal regression from the fMRI preprocessing pipeline.

### 2.5 Statistical analysis

Statistical analyses were conducted using SPSS (version 25). Demographic and clinical data were compared between groups using one-way analysis of variance (ANOVA), independent samples t-tests and Mann-Whitney U tests. Sex differences were assessed using the Chi-square test. Group differences in Crus II volume and FC were examined through analysis of covariance (ANCOVA), with age, sex, and education level as key covariates due to their established impact on brain structure and cognitive function (Diedrichsen et al., 2009, Hou et al., 2019). To ensure the robustness of our findings, sensitivity analyses were conducted within nonFA and FA groups by repeating the ANCOVAs with lesion volume and disease duration as additional covariates. Following significant main effects, Bonferroni-corrected post hoc pairwise comparisons were conducted. Furthermore, we conducted partial correlation analyses. These analyses focused exclusively on the neuroimaging metrics that had demonstrated significant group differences (R_MDTB7/8/9-LN FC and R_Crus II volume), and their association with AQ. To account for this focused set of correlations, the FDR correction was applied. All analyses were conducted separately for each group, controlling for age, sex, education level, lesion volume, and disease duration. To formally test for subtype-specific effects, direct comparisons of correlation coefficients between the nonFA and FA groups were conducted using Fisher’s *r*-to-*z* transformations.

To investigate the interplay between volume, FC and language scores, we performed mediation and moderated mediation analyses using PROCESS macro (version 3.5) for SPSS (version 25). Prior to mediation analysis, the required pathway associations (predictor-outcome, predictor-mediator, and mediator-outcome adjusted for predictor) were first established using hierarchical linear regression, controlling for age, sex, education level, lesion volume and disease duration. Multicollinearity among predictors was ruled out (variance inflation factors < 3.0). For simple mediation effects, Model 4 was employed to estimate the indirect path. To further test whether the mediation process was conditional on group (nonFA vs FA), we utilized Model 8, which allowed the group to influence both the direct path and the first stage of the indirect path. Mediation and moderated mediation effects were estimated using a bootstrapping procedure (5,000 samples) to derive 95% confidence intervals (CI) for the direct, indirect, and conditional indirect pathways. The moderated mediation was considered statistically significant if the 95% CI of the Index of Moderated Mediation did not include zero, indicating that the indirect effect significantly differed across groups. To ensure clarity regarding the specific covariates included in each distinct analysis, a detailed breakdown is provided in Supplementary Table S3.

## 3 Results

### 3.1 Demographic and clinical features in primary cohort

The primary cohort included 81 PSA patients (mean age 56.10 ± 11.26 years, range 29–79; 32.10% female) and 77 HCs (mean age 55.70 ± 11.44 years, range 29–77; 35.06% female). The groups did not significantly differ in terms of sex distribution (*p* = 0.229) or education level (*p* = 0.08). When patients were classified into FA (n = 35; mean age 51.66 ± 11.67 years, range 29–72; 31.43% female) and nonFA (n = 46; mean age 59.48 ± 9.77 years, range 38–79; 32.61% female), significant differences in age (*F_(2,155)_* = 5.02, *p* = 0.008) and education level (*F_(2,155)_* = 3.93, *p* = 0.022) were observed among the three groups, but did not differ significantly in sex (*p* = 0.919).

As expected, the nonFA group presented with lower fluency scores and AQ compared to FA (all *p* < 0.001). Within the patient subgroups, there were no differences in sex, disease duration, or total lesion volume (all *p* > 0.05). However, the nonFA group was older (*p* = 0.002) and had a lower education level (*p* = 0.017). Consequently, age and education were treated as covariates in all subsequent analyses. Demographic and clinical details are provided in Table 1. As a further step to ensure the results were not driven by these or other potential confounds, a series of sensitivity analyses were performed to specifically assess the impact of demographic variables, head motion, and preprocessing choices.

### 3.2 FC analyses

**Fig. 3:**
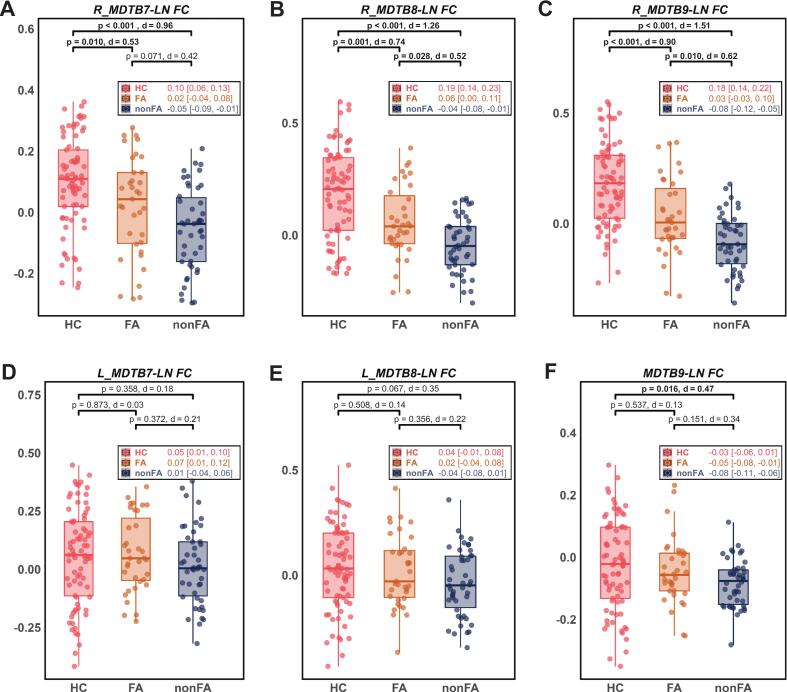
Differences among groups in functional connectivity. Group comparison of (A) R_MDTB7-LN FC (*F_(2,152)_* = 13.27, *p* < 0.001, *η^2^* = 0.15), (B) R_MDTB8-LN FC (*F_(2,152)_* = 22.94, *p* < 0.001, *η^2^* = 0.23), (C) R_MDTB9-LN FC (*F_(2,152)_* = 32.80, *p* < 0.001, *η^2^* = 0.30), (D) L_MDTB7-LN FC (*F_(2,152)_* = 0.53, *p* = 0.588, *η^2^* = 0.01), (E) L_MDTB8-LN FC (*F_(2,152)_* = 1.71, *p* = 0.185, *η^2^* = 0.02) and (F) MDTB9-LN FC (*F_(2,152)_* = 2.95, *p* = 0.055, *η^2^* = 0.04) across three groups, using ANCOVA (age, sex and education level as covariates). Post-hoc pairwise comparisons utilized Bonferroni-corrected. Bold font indicates statistical significance (*p* < 0.05, Bonferroni-corrected). Box plots display medians (central lines) and IQRs (box boundaries), with overlaid scatter points showing individual data. Inset labels provide group means and 95% CIs. Brackets indicate post-hoc *p*-values and Cohen’s *d*. Abbreviations: ANCOVA = Analysis of covariance; MDTB = Multi-Domain Task Battery; LN = classical language network; FC = functional connectivity; FA = fluent aphasia; nonFA = non-fluent aphasia; L/R_MDTB7/8/9-LN FC = FC between the language network and peak coordinates in left/right MDTB7/8/9; IQR = interquartile range; CI = confidence interval.

Initial whole-parcel analysis, with age, sex, education as covariates, revealed a trend-level group difference in FC between LN and the MDTB9 subregion (*F_(2,152)_* = 2.95, *p* = 0.055, *η^2^* = 0.04), subsequently, the post-hoc comparisons confirmed significant differences between the nonFA and HC (*p_bonf_* = 0.016, Cohen’s *d* = 0.47) (Fig. 3, Supplementary Tables S4 and S5). No group effects were observed for the MDTB7 or MDTB8 parcels (Supplementary Fig. S1).

In contrast, patient-specific peak connectivity analyses unveiled a more robust and specific pattern. Highly significant group effects emerged for FC between the LN and peak coordinates within the MDTB7 (R_MDTB7-LN FC; *F_(2,152)_* = 13.27, *p* < 0.001, *η^2^* = 0.15), MDTB8 (R_MDTB8-LN FC; *F_(2,152)_* = 22.94, *p* < 0.001, *η^2^* = 0.23), and MDTB9 (R_MDTB9-LN FC; *F_(2,152)_* = 32.80, *p* < 0.001, *η^2^* = 0.30). Post-hoc comparisons between patient subgroups confirmed these effects for R_MDTB8-LN FC (*p_bonf_* = 0.028, Cohen’s *d* = 0.52) and R_MDTB9-LN FC (*p_bonf_* = 0.010, Cohen’s *d* = 0.62), while the R_MDTB7-LN FC effect was no longer statistically significant (*p_bonf_* = 0.071, Cohen’s *d* = 0.42) (Fig. 3A-C, Supplementary Table S5). This right-lateralized pattern was underscored by the absence of any group effects for the homologous left-hemisphere peak coordinates (all *p* > 0.05) (Fig. 3D-E; Supplementary Table S4 and S5).

Sensitivity analysis between FA and nonFA subgroups, which additionally controlled for lesion volume and disease duration, confirmed the persistent significance for R_MDTB8-LN FC (*F_(1,74)_* = 7.47, *p* = 0.008, *η^2^* = 0.09) and R_MDTB9-LN FC (*F_(1,74)_* = 6.07, *p* = 0.016, *η^2^* = 0.08), whereas the R_MDTB7-LN FC effect was attenuated to non-significance (*F_(1,74)_* = 2.70, *p* = 0.105, *η^2^* = 0.04) (Supplementary Table S4).

Finally, to directly compare the utility of our individualized approach against the atlas-based centroids method, we performed a hierarchical regression analysis. As detailed in Supplementary Table S6, the individualized peak FC consistently explained significant additional variance in AQ scores beyond the atlas-based centroids model across all ROIs. Specifically, the individualized predictor for MDTB7 accounted for an additional 8% of variance (Δ*R^2^* = +0.08, *F*-change = 8.94, *p* = 0.004), with a corresponding improvement in model fit (ΔAIC = +7.41). The effect was even more pronounced for MDTB8, explaining an additional 11% of variance (Δ*R^2^* = +0.11, *F*-change = 13.13, *p* = 0.001) and substantially improving the model (ΔAIC = +14.05). A significant advantage was also observed for MDTB9 (Δ*R^2^* = +0.04, *F*-change = 4.79, *p* = 0.032; ΔAIC = +2.20).

### 3.3 Volumetric analyses

Individualized FC analyses identified a notable spatial convergence, with peak coordinates in MDTB 7, 8, and 9 consistently mapping to the right Crus II lobules. This data-driven finding provided a strong anatomical rationale to test for corresponding structural differences in this specific cerebellar subregion.

**Fig. 4:**
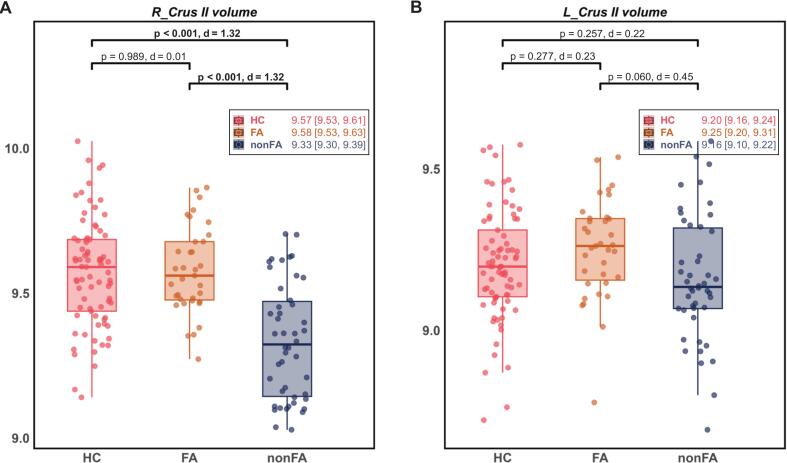
Differences among groups in Crus II volume. Group comparison of (A) R_Crus II (*F_(2,152)_* = 25.98, *p* < 0.001, *η^2^* = 0.26) and (B) L_Crus II volume (*F_(2,152)_* = 1.81, *p* = 0.168, *η^2^* = 0.02) across three groups, using ANCOVA (age, sex and education level as covariates). Post-hoc pairwise comparisons utilized Bonferroni-corrected. Bold font indicates statistical significance (*p* < 0.05, Bonferroni-corrected). Box plots display medians (central lines) and IQRs (box boundaries), with overlaid scatter points showing individual data. Inset labels provide group means and 95% CIs. Brackets indicate post-hoc *p*-values and Cohen’s *d*. Abbreviations: ANCOVA = Analysis of covariance; FA = fluent aphasia; nonFA = non-fluent aphasia; L/R_Crus II = left/right Crus II; IQR = interquartile range; CI = confidence interval.

ANCOVA modeling, incorporating age, sex, and education level as covariates, unveiled a significant group difference in R_Crus II volume (*F_(2,152)_* = 25.98, *p* < 0.001, *η^2^* = 0.26), primarily attributed to differences among the PSA subgroups in subsequent post-hoc analyses (*p* = 0.024, Bonferroni-corrected) (Fig. 4A and Supplementary Table S4 and S5). Mirroring the lateralization of our FC findings, no such group difference was observed in L_Crus II volume (*F_(2,152)_* = 1.81, *p* = 0.168, *η^2^* = 0.02) (Fig. 4B).

This right-lateralized volumetric effect remained robust, with a sensitivity analysis confirming a highly significant difference between the PSA subgroups even after controlling for lesion volume and disease duration (*F_(1,74)_* = 24.39, *p* < 0.001, *η^2^* = 0.25).

### 3.4 Correlations between Functional/Structural indicators and language performance

Given that the AQ offers a comprehensive measure of aphasia severity, and its availability in the public ARC dataset was critical for ensuring consistency in our replication analysis, it was selected as the primary behavioral variable for correlation analyses.

**Fig. 5:**
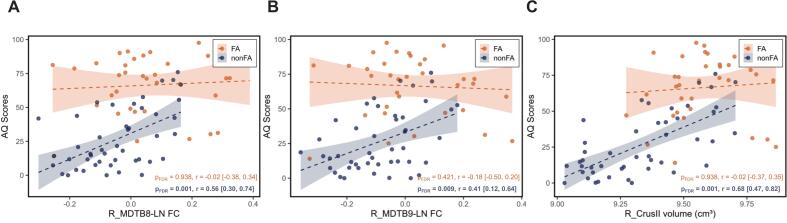
Associations between functional/structural indicators and language performance. Correlation between (A) R_MDTB8-LN FC, (B) R_MDTB9-LN FC, (C) R_Crus II volume and AQ scores. Solid line ± shaded 95% confidence band depicts rank-based regression; *r* and FDR-corrected *p*-values are annotated; Bold font indicates statistical significance (*p_FDR_* < 0.05). Fisher’s r-to-z comparisons confirmed that brain-behavior correlations were significantly stronger in the nonFA group than in the FA group for R_MDTB8-LN FC (*Z* = −2.62, *p* = 0.009), R_MDTB9-LN FC (*Z* = −2.64, *p* = 0.008), and R_CrusII volume (*Z* = −3.43, *p* < 0.001). Abbreviations: AQ = Aphasia Quotient; FC = functional connectivity; FA = fluent aphasia; nonFA = non-fluent aphasia; MDTB = Multi-Domain Task Battery; LN = classical language network; L/R_MDTB7/8/9-LN FC = FC between the language network and the peak coordinates in the left/right MDTB7/8/9; L/R_Crus II = left/right Crus II.

Partial correlation analyses showed that, in the nonFA group, higher AQ scores were significantly correlated with increased R_MDTB8-LN FC (*r* = 0.56 [0.30, 0.74], *p_FDR_* < 0.001) and R_MDTB9-LN FC (*r* = 0.41 [0.12, 0.64], *p_FDR_* = 0.009), and larger R_Crus II volume (*r* = 0.68 [0.47, 0.82], *p_FDR_* = 0.001). In stark contrast, these correlations were absent in the FA group, where no markers correlated with AQ (all *p_FDR_* > 0.05; Fig. 5). To formally test this apparent dissociation, Fisher’s *r*-to-*z* transformations were performed. The analysis confirmed that the correlations were indeed significantly stronger in the nonFA group for R_MDTB8-LN FC (*Z* = −2.62, *p* = 0.009), R_MDTB9-LN FC (*Z* = −2.64, *p* = 0.008), and R_CrusII volume (*Z* = −3.43, *p* < 0.001).

Neither R_MDTB7-LN FC (*r* = 0.25 [-0.06, 0.22], *p_FDR_* = 0.136) nor MDTB9-LN FC (*r* = −0.08 [-0.39, 0.24], *p_FDR_* = 0.620) significantly correlated with AQ in the nonFA group, despite both metrics showing significant group differences (Supplementary Fig. S2). These associations were likewise absent in the FA group (all *p_FDR_* > 0.05). All relevant correlations for the different indicators are visually presented in Supplementary Fig. S3, with all uncorrected *p*-values meticulously listed in Supplementary Tables S7 and S8.

### 3.5 Mediation and moderated mediation analyses

**Fig. 6:**
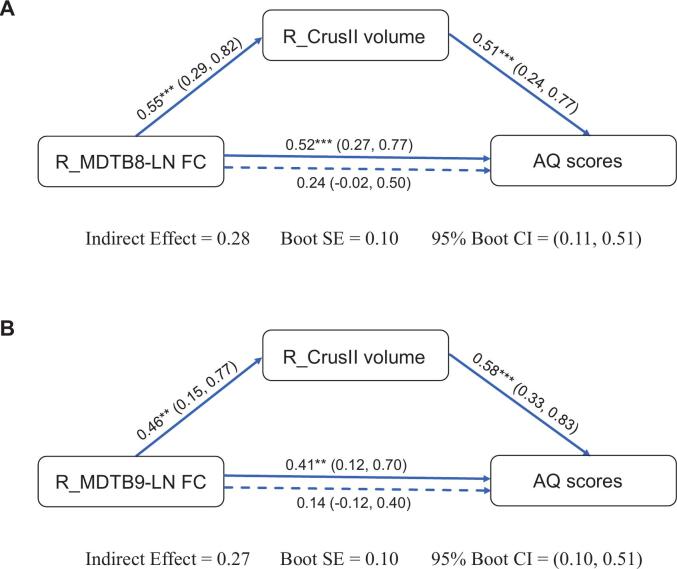
Mediation models: Associations among R_MDTB8/9-LN FC, R_Crus II volume, and AQ scores in the nonFA group. R_Crus II volume as a mediator between cerebellar FC and AQ for (A) R_MDTB8-LN FC and (B) R_MDTB9-LN FC. All paths display standardized regression coefficients (*β*) with 95% confidence intervals. The total effect is shown above the direct effect (dashed). The indirect effects for both models were significant, confirmed via bootstrapping (5,000 samples), with the 95% bootstrapped confidence intervals excluding zero. **p < 0.01, ***p < 0.001. Abbreviations: AQ = Aphasia Quotient; FC = functional connectivity; FA = fluent aphasia; nonFA = non-fluent aphasia; MDTB = Multi-Domain Task Battery; LN = classical language network; L/R_MDTB7/8/9-LN FC = FC between the language network and the peak coordinates in the left/right MDTB7/8/9; L/R_Crus II = left/right Crus II.

Mediation analysis within the nonFA group to explore the interplay between R_MDTB8/9-LN FC, R_Crus II volume, and AQ scores, adjusting for potential confounders (age, sex, education level, lesion volume and disease duration). After establishing the prerequisite associations (Supplementary Tables S9 and S10), robust mediation analysis with 5,000 bootstraps demonstrated significant indirect effects of R_Crus II volume on the relationship between R_MDTB8-LN FC and AQ scores (*β* = 0.28, SE = 0.10, 95% CI [0.12, 0.51]) (Fig. 6A and Supplementary Table S11) and R_MDTB9-LN FC and AQ scores (*β* = 0.27, SE = 0.10, 95% CI [0.09, 0.51]) (Fig. 6B and Supplementary Table S12). Direct effects of cerebello-cortical FC pathways on AQ scores became non-significant when controlling for R_Crus II volume (R_MDTB8-LN FC → AQ: *β* = 0.24, SE = 0.13, 95% CI [-0.02, 0.50]; R_MDTB9-LN FC → AQ: *β* = 0.14, SE = 0.13, 95% CI [-0.12, 0.40]), suggesting that R_Crus II structural preservation largely accounts for the statistical association observed between enhanced FC and higher AQ scores.

To test whether the indirect effects were conditional upon group, we conducted moderated mediation analyses using PROCESS Model 8. The results revealed a significant index of moderated mediation, indicating that the indirect effect of R_MDTB8-LN FC on AQ through R_Crus II volume was significantly moderated by group (Index = 0.19, SE = 0.11, 95% CI [0.03, 0.44]; Supplementary Fig. S4A). And the indirect effect of R_MDTB9-LN FC on AQ via R_Crus II volume was also contingent on group membership (Index = 0.21, SE = 0.11, 95% CI [0.02, 0.45]; Supplementary Fig. S4B). As the confidence intervals for both indices exclude zero, these results collectively support the hypothesis that the strength of both mediational pathways differs significantly between the two groups.

To further explore the robustness of our statistical findings, we tested an alternative mediation model by reversing the roles of the initial independent variable and mediator. Specifically, we examined whether the R_MDTB8/9-LN FC mediated the relationship between right Crus II volume and AQ scores. This alternative pathway failed to reach statistical significance (R_Crus II volume → R_MDTB8-LN FC → AQ: *β* = 0.05, SE = 0.05, 95% CI [-0.05, 0.17]; R_Crus II volume → R_MDTB9-LN FC → AQ: *β* = 0.01, SE = 0.04, 95% CI [-0.05, 0.10]; Supplementary Table S13), which is consistent with the observed pattern in our primary mediation analysis.

### 3.6 Sensitivity analysis

#### 3.6.1 Robustness of key findings in a demographically matched subsample

To confirm our findings were robust against demographic confounds, we performed a sensitivity analysis on a demographically matched subsample (75 HCs, 29 FA, 41 nonFA), which showed no significant group differences in age, sex, or education (all *p* > 0.05; Supplementary Table S14A).

The results were highly consistent with the primary analysis. For instance, the significant group differences in R_MDTB8-LN FC (*F_(2,139)_* = 21.22, *p* < 0.001, partial *η^2^* = 0.23), R_MDTB9-LN FC (*F_(2,139)_* = 31.46, *p* < 0.001, partial *η^2^* = 0.31), and R_Crus II volume (*F_(2,139)_* = 22.84, *p* < 0.001, partial *η^2^* = 0.25) all remained highly significant in the matched subsample. The stability of these effects was further confirmed in an ANCOVA between FA and nonFA patient groups that also controlled for lesion volume and disease duration (Supplementary Table S14B).

#### 3.6.2 Robustness to preserved LN volume as a covariate

To confirm the robustness of our findings, we conducted a sensitivity analysis by replacing lesion volume with preserved LN volume as the covariate. The results were highly consistent with our primary analyses. Specifically, the significant group differences in functional connectivity were maintained for both R_MDTB8-LN (*F_(1,74)_* = 8.61, *p* = 0.004, partial *η^2^* = 0.10) and R_MDTB9-LN (*F_(1,74)_* = 7.14, *p* = 0.009, partial *η^2^* = 0.09) (Supplementary Table S15A). Furthermore, the critical correlations with aphasia severity in the non-fluent group remained significant for both R_MDTB8-LN FC (*r* = 0.53 [0.26, 0.72], *p_FDR_* = 0.001) and R_MDTB9-LN FC (*r* = 0.39 [0.09, 0.62], *p_FDR_* = 0.022) (Supplementary Table S15B). Finally, the key indirect effects in our mediation models also held, with significant pathways through R_Crus II for both R_MDTB8-LN (*β* = 0.25, SE = 0.06, 95% CI [0.13, 0.39]) and R_MDTB9-LN (*β* = 0.19, SE = 0.06, 95% CI [0.08, 0.34]) pathways (Supplementary Table S15C).

#### 3.6.3 Robustness to total intracranial volume

We conducted a sensitivity analysis where TIV was included as an additional covariate in our ANCOVA models. The significant group differences in R_Crus II volume observed in our primary analyses remained highly consistent even after controlling for TIV (Supplementary Table S16). For the comparison across HC, FA, and nonFA groups, a significant effect was observed (*F_(2, 149)_* = 25.89, *p* < 0.001, partial *η^2^* = 0.26), and for the comparison between FA and nonFA groups, the significant effect remained (*F_(1, 75)_* = 24.13, *p* < 0.001, partial *η^2^* = 0.25). These findings underscore that the observed group differences in right Crus II volume are robust and independent of individual variations in total intracranial volume.

#### 3.6.4 Robustness to stricter head motion control

To confirm the robustness of our findings, we conducted a sensitivity analysis by restricting the sample to participants with a mean framewise displacement (FD) of less than 0.2 mm. The results were highly consistent with our primary analyses (Supplementary Table S17). Specifically, the significant group differences in functional connectivity between HC, FA, and nonFA groups were maintained for R_MDTB7-LN FC (*F_(2, 134)_* = 12.62, *p* < 0.001, partial *η^2^* = 0.16), R_MDTB8-LN FC (*F_(2, 134)_* = 22.00, *p* < 0.001, partial *η^2^* = 0.25) and R_MDTB9-LN FC (*F_(2, 134)_* = 27.16, *p* < 0.001, partial *η^2^* = 0.29).

#### 3.6.5 Robustness to global signal regression

We performed another sensitivity analysis by omitting global signal regression (GSR) from the preprocessing pipeline, using the same low-motion subsample (mean FD < 0.2 mm). The results were largely consistent with both our primary analyses and the stricter head motion control analysis (Supplementary Table S17). Specifically, significant group differences between HC, FA, and nonFA groups were still evident for MDTB9-LN FC (*F_(2, 134)_* = 5.88, *p* = 0.004, partial *η^2^* = 0.08), R_MDTB7-LN FC (*F_(2, 134)_* = 8.86, *p* < 0.001, partial *η^2^* = 0.12), R_MDTB8-LN FC (*F_(2, 134)_* = 18.12, *p* < 0.001, partial *η^2^* = 0.22), and R_MDTB9-LN FC (*F_(2, 134)_* = 13.93, *p* < 0.001, partial *η^2^* = 0.18).

### 3.7 External validation in an independent ARC cohort

**Fig. 7:**
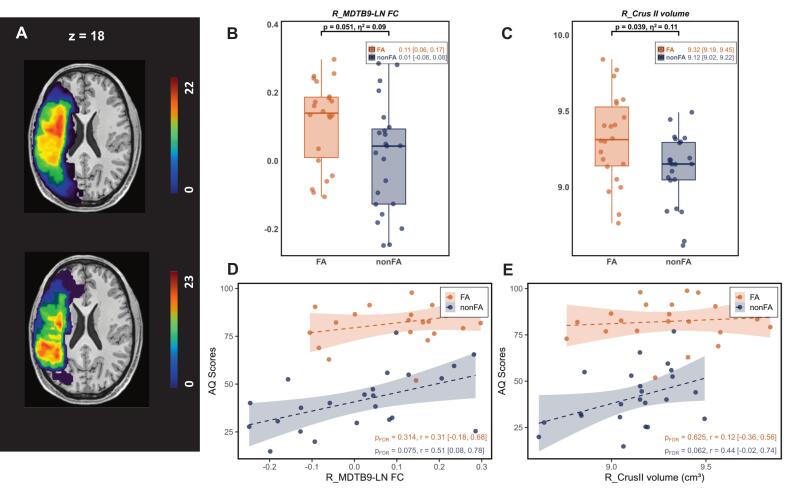
Lesion-overlap and Verification results in the ARC. (A) Lesion-overlap heat-map for the FA (n = 22, the above) and the nonFA (n = 23, the below) groups; warmer colors denote a higher proportion of subjects with damage to a given voxel; Color bar indicates the number of patients. Group comparison of (B) R_MDTB9-LN FC (*F_(1,39)_* = 4.06, *p* = 0.051, *η^2^* = 0.09) and (C) R_Crus II volume (*F_(1,39)_* = 4.57, *p* = 0.039, *η^2^* = 0.11) between FA and nonFA, using ANCOVA (age, sex, lesion volume and disease duration as covariates). *P*-values and effect sizes (*η^2^*) are displayed above the plots. Mean values and 95% confidence intervals are provided for each group. (D) Correlation between R_MDTB9-LN FC and AQ. (E) Correlation between R_Crus II volume and AQ. Solid line ± shaded 95% confidence band depicts rank-based regression; *r* and FDR-corrected *p-*values are annotated; Bold font indicates statistical significance (*p*_FDR_ < 0.05). Abbreviation: AQ = Aphasia Quotient; FC = functional connectivity; FA = fluent aphasia; nonFA = non-fluent aphasia; MDTB = Multi-Domain Task Battery; LN = classical language network; L/R_MDTB7/8/9-LN FC = FC between the language network and the peak coordinates in the left/right MDTB7/8/9; L/R_Crus II = left/right Crus II. Z refer to the z-plane coordinates of the MNI space. Color bar indicates the number of patients.

We included 45 PSA patients from the ARC database and classified them into FA (n = 22; mean age 62.64 ± 11.85 years, range 27–78; 45.45% female) and nonFA (n = 23; mean age 58.91 ± 12.71 years, range 36–76; 47.83% female). Age, sex, and disease duration did not differ significantly between subgroups (all *p* > 0.05), whereas lesion volume (*p* < 0.001) and AQ (*p* < 0.001) differed markedly. Demographics and clinical variables are detailed in Table 2; the lesion-overlap map is depicted in Fig. 7A.

We first tested for the group differences in our key neuroimaging markers, with age, sex, lesion volume and disease duration as covariates. This analysis partially replicated our findings: we observed a strong trend for R_MDTB9-LN FC (*F_(1,39)_* = 4.06, *p* = 0.051, *η^2^* = 0.09) and R_Crus II volume (*F_(1,39)_* = 4.57, *p* = 0.039, *η^2^* = 0.11), though these did not survive correction, while R_MDTB8-LN FC (*F_(1,39)_* = 0.36, *p* = 0.550, *η^2^* = 0.01) did not differ significantly (Fig. 7B-C, Supplementary Fig. S5A).

Partial correlation analyses revealed a notable finding exclusively in the nonFA group. Specifically, R_MDTB9-LN FC showed a significant positive correlation with AQ at an uncorrected threshold (*r* = 0.51 [0.08, 0.78], *p* = 0.025; Fig. 7D). While this association did not survive strict FDR correction (*p_FDR_* = 0.075), it nonetheless represented a strong, uncorrected trend. This pattern was further supported by another trend-level correlations observed only in the nonFA group: the R_Crus II volume with AQ (*r* = 0.44 [-0.02, 0.74], *p_FDR_* = 0.062; Fig. 7E). As expected, these brain-behavior relationships were entirely absent in the FA group (all *p* > 0.05; Fig. 7D-E), highlighting the specificity of our findings.

## 4 Discussion

This study provides novel insights into the neural mechanisms underlying language fluency in PSA. Our results demonstrate that patients with nonFA exhibited significantly lower FC between language-related cerebellar subregions and preserved LN, alongside reduced right Crus II volume, compared FA and HCs. Critically, within nonFA patients, these indicators are positively correlated with language performance, while this relationship is absent in FA. Furthermore, our analysis revealed that the volume integrity of the right Crus II statistically accounts for the relationship observed between cerebello-LN FC and language performance. These findings establish specific cerebellar subregions as critical nodes within the neural mechanisms of language fluency in PSA and position them as promising targets for future therapeutic interventions.

Previous research has emphasized the critical role of the right hemisphere of cerebellum in language (Lesage et al., 2017, Mariën et al., 1996). In line with these findings, we observed that only right-hemispheric cerebellar indicators showed significant differences and a positive correlation with language performance, while no such associations were found for the left cerebellum. The observed cerebellar alterations are interpreted not as independent primary findings, but rather as secondary, adaptive, or potentially maladaptive neuroplastic changes resulting from the primary left-hemispheric cortical lesions. The crossed cerebello-cerebral theory posits that the right cerebellum connects with the left-hemispheric language network via contralateral projections (Marien et al., 2001). Following left-hemispheric stroke, this connection is disrupted (Stilling et al., 2025), consistent with our observation of reduced R_MDTB 7/8/9-LN FC in the overall patient group. The neuroimaging indictors (FC/volume) and circuit function can be influenced by altered tissue properties resulting from cellular immune responses to brain injury, including neuroglial activation, peripheral immune cell infiltration, and the release of cytokines and chemokines (Anrather and Iadecola, 2016). These inflammatory processes could modulate network activity and contribute to the observed changes. Another explanation of the structural atrophy in the right Crus II may be caused by crossed cerebellar diaschisis (CCD). CCD is the phenomenon of decreased blood flow and metabolism within the contralateral cerebellum after supratentorial structural impairment (Yamauchi et al., 1992, Reivich, 1992). It has been reported that patients with CCD may further experience cerebellar atrophy (Tien and Ashdown, 1992). Our results provide strong clinical support to the concept of a right-lateralized linguistic cerebellum, underscoring the right cerebellum’s integral contribution to the broader networks that maintain language function.

Beyond simply confirming this lateralization, our findings provided strong clinical support for the cerebellum’s role in linguistic fluency, by singling out the nonFA group, which presented with more severe reductions in cerebellar FC and volume compared with FA and HCs. This distinction was further highlighted by the finding that a positive correlation between these indicators and language performance was present exclusively in nonFA patients. The well-established functions of cerebellum include sequencing, timing, and error correction, all of which are critical components of fluent speech (Sokolov et al., 2017, Argyropoulos, 2016). Notably, our study highlights the cerebellum’s role in speech motor control and planning, distinguishing nonFA from symptomatically similar disorders like ataxic dysarthria. Ataxic dysarthria, emerging directly from cerebellar lesions, is classically viewed as a deficit of speech motor coordination, manifesting as unclear articulation and abnormal prosody (Spencer and Slocomb, 2007). In contrast, non-fluent aphasia, stemming from left-hemispheric lesions, is primarily considered a deficit of linguistic and speech motor planning, characterized by laborious, agrammatic output (Riccardi et al., 2024). This functional overlap underscores the cerebellum’s integral contribution to the broader networks that support fluent speech.

The core contribution of this study is the identification of functionally-defined specific subregions of the cerebellum, which are critical to this process. Significant alterations were largely confined to the connections between MDTB8/9 and LN, indicating a selective involvement of these subregions rather than a diffuse cerebellar dysfunction in nonfluent aphasia. Previous studies have often treated the cerebellum as a unitary structure or relied solely on anatomical atlases. Building upon the MDTB functional atlas proposed by King et al. (King et al., 2019), which delineates a high degree of specialization within the cerebellum, our study is the first to apply this divisions to patients with PSA. Within this framework, MDTB9 is strongly associated with verbal fluency and broader language processing, suggesting a role in lexical selection and the sequential organization of linguistic units during speech production. MDTB8, characterized by features related to word comprehension and narrative processing, may contribute to internal linguistic representations and monitoring processes that detect mismatches between intended and produced speech (Mechtenberg et al., 2024). Our findings extend previous work by Stilling et al. (Stilling et al., 2025), demonstrating that while naming and fluency share overlapping cerebellar substrates, fluency engages broader MDTB-defined regions and likely requires integrated operations of lexical selection, sequencing, and predictive monitoring. Consistent with these functional interpretations, our strongest connectivity peaks localized to the right cerebellar Crus II across aphasia subtypes, suggesting that this region acts as a stable cerebellar node within language-related networks despite variability in cortical lesion locations. Empirically, we observed that in nonFA, R_MDTB8/9-LN FC and right Crus II volume were positively correlated with language performance, whereas these relationships were absent in FA. Direct statistical comparison confirmed that the correlations were significantly stronger in the nonFA group. This increased functional coupling may reflect a compensatory mechanism that helps sustain language performance within the limits imposed by the existing cortical damage (Alho et al., 2023). These findings demonstrate that language recovery after stroke may depend on highly specialized cerebellar subregions and their interaction with cortical language networks. They further suggest that targeted modulation of the cerebello-LN circuit could represent a promising strategy to enhance neural plasticity and improve post-stroke language outcomes (Jossinger et al., 2024).

Our findings have significant clinical and translational implications. We suggest that the FC strength of the R_MDTB8/9-LN and the volume of the right Crus II show promise as neuroimaging biomarkers for prognosticating language outcomes in PSA (especially nonFA). We pioneered the application of the MDTB functional atlas in PSA research, enabling a functionally nuanced exploration of the cerebellum’s fine-grained division of labor that transcends the limits of traditional anatomical boundaries, suggesting that future non-invasive neuromodulation protocols could consider targeting specific language-related cerebellar subregions as potential targets. Moreover, the application of a mediation analysis model enabled a deeper inquiry into the interplay between cerebello-LN FC, volume, and language function in nonFA, suggesting a structure–function-behavior pathway supporting fluent speech (Strick et al., 2009), moving beyond simple correlations. Additionally, our study employ individualized, lesion-dependent mapping to identify peak locus between the functionally-defined cerebellar subregions and preserved LN, surpassing the limitations of conventional anatomical boundaries and group-level analyses (Stefaniak et al., 2022), supporting targeted, patient-centric therapeutic strategies (Yu et al., 2017). Finally, the inclusion of an independent external validation cohort significantly enhances the robustness and generalizability of our conclusions. Although some results reached only trend-level significance, the partial replication of our core findings in this independent dataset lends considerable stability to our results.

Several limitations should be considered. Our cross-sectional design, although effective for identifying associations, cannot establish temporal precedence. Our study implies that the model illustrates statistical associations among cerebello-LN connectivity, right Crus II volume, and language performance, without establishing causality. Longitudinal studies tracking patients from the acute post-stroke phase through recovery are necessary to confirm the proposed mechanistic sequence. Additionally, we did not conduct split-half reliability assessments or longitudinal test–retest analyses. Future research would benefit from longer scan acquisitions, as patient tolerance allows, or from utilizing multi-timepoint longitudinal data to better characterize the dynamic stability of these cerebellar-cortical functional circuits. While our statistical tests confirmed stronger correlations in the nonFA group, group differences in data variance warrant consideration. The FA group’s higher and more clustered AQ scores suggest a potential ceiling effect, which may have attenuated the correlations observed in this subtype. Future studies with a wider range of language severity in fluent aphasia are needed to corroborate this finding. Furthermore, the use of atlas-based regions, even when optimized with SUIT, does not fully account for individual anatomical variability. Importantly, the lesion-constrained mapping strategy used to construct the healthy reference introduces an additional consideration, as the derived baseline is influenced by patient lesion geometry and may not fully reflect an intact language network. Lastly, our original clinical protocol lacked targeted behavioral assessments for apraxia of speech or dysarthria; future studies should diligently incorporate such detailed evaluations to specifically assess and potentially control for the confounding influence of these motor-speech factors. Future studies should explore several complementary avenues to extend and refine our findings. Advanced neuroimaging techniques, such as Diffusion Tensor Imaging (DTI), could further elucidate the microstructural integrity of the specific white matter tracts underpinning the cerebello-thalamo-cortical loop. Our study focuses on the dimension of fluency. Future research will expand to other dimensions to explore the role of cerebellar subregions in different language dimensions.

## 5 Conclusion

In conclusion, our findings demonstrate that FC strength and volume integrity of language-related cerebellar subregions contribute to fluency. Results support expanding PSA models beyond cortical regions and suggest targeting the cerebello-LN circuit with neuromodulation strategies may increase the plasticity of the residual cortical language network and subsequently improve rehabilitation language outcomes.

## CRediT authorship contribution statement

**Yuqian Zhan:** Writing – original draft, Software, Project administration, Methodology, Investigation, Formal analysis, Conceptualization. **Xiaohui Xie:** Writing – review & editing, Software, Methodology, Investigation, Formal analysis, Conceptualization. **Qiufang Ren:** Writing – review & editing, Software, Investigation, Formal analysis. **Xiaomin Pan:** Writing – review & editing, Supervision, Methodology. **Zhishun Gao:** Writing – review & editing, Supervision. **Jin Li:** Writing – review & editing, Supervision. **Kai Wang:** Writing – review & editing, Supervision, Project administration, Methodology, Funding acquisition, Conceptualization. **Tongjian Bai:** Writing – review & editing, Supervision, Project administration, Methodology, Funding acquisition, Conceptualization. **Panpan Hu:** Writing – review & editing, Supervision, Project administration, Methodology, Funding acquisition, Conceptualization.

## Funding

This work was supported by the 10.13039/501100001809National Natural Science Foundation of China (grant numbers: 82171917, 82471271, U23A20424).

## Declaration of competing interest

The authors declare that they have no known competing financial interests or personal relationships that could have appeared to influence the work reported in this paper.

## Data Availability

Data will be made available on request.
