# Supplementary material for: Beyond the cerebral cortex: cerebellar language-related subregions contributions to fluency in post-stroke aphasia
Source: Neuroimage Clin. 2026 Apr 29;50:103999. doi: 10.1016/j.nicl.2026.103999 (PMC13158568; doi:10.1016/j.nicl.2026.103999)
Supplement: Supplementary Data 1 [file mmc1.docx]

**Supplemental materials**

**Beyond the cerebral cortex: Cerebellar language-related subregions contributions to fluency in post-stroke aphasia**

**Authors:**

Yuqian Zhan ^a, *^; Xiaohui Xie ^b, *^; Qiufang Ren ^a, *^; Xiaomin Pan ^a^; Zhishun Gao ^a^; Jin Li ^a^; Kai Wang ^a,c,d,e, #^; Tongjian Bai ^a, #^; Panpan Hu ^a, #^

^a^ Department of Neurology, The First Affiliated Hospital of Anhui Medical University, Anhui Medical University, Hefei 230032, China;

^b^ Department of Neurology, The Second Affiliated Hospital of Anhui Medical University, Anhui Medical University, Hefei 230601, China;

^c^ Anhui Institute of Translational Medicine, Hefei 230032, China;

^d^ Institute of Artificial Intelligence, Hefei Comprehensive National Science Center, Hefei 230032, China;

^e^ Anhui Province Key Laboratory of Cognition and Neuropsychiatric Disorders, Hefei 230032, China;

^*^ Yuqian Zhan, Xiaohui Xie and Qiufang Ren equally contributed to this work.

^#^ **Authors for correspondence:** Kai Wang, E-mail: [wangkai1964@126.com](mailto:wangkai1964@126.com); Tongjian Bai, E-mail: [baiyunong1990@163.com](mailto:baiyunong1990@163.com). Panpan Hu, E-mail: [hupanpan@ahmu.edu.cn](mailto:hupanpan@ahmu.edu.cn)

**Address:** No. 218 Jixi Road, Shushan District, Hefei, Anhui, China

**Supplemental Tables**

**Supplementary Table S1. Comparison between primary and validation (ARC) cohorts.**

| Characteristics | Primary Cohort | Validation Cohort (ARC) |
| --- | --- | --- |
| Sample Size | FA: n = 35; nonFA: n = 46; HC: n = 77 | FA: n = 22; nonFA: n = 23 |
| MRI Acquisition Site | The University of Science and Technology of China | The University of South Carolina |
| MRI Scanner | 3.0T GE Discovery 750 | 3.0T Siemens Trio |
| T1-weighted MRI Parameters | TR/TE: 8.16/3.18 ms; TI: 450 ms;  Flip Angle: 12°; Voxel Size: 1 × 1 × 1 mm^3^ | TR/TE: 2250/4.15 ms; TI: 925 ms;  Flip Angle: 9°; Voxel Size: 1 × 1 × 1 mm^3^ |
| Rs-fMRI Parameters | TR/TE: 2400/30 ms; Flip Angle: 90°; Slice Thickness: 3 mm;  Volumes: 217 | TR/TE: 1650/35 ms; Flip Angle: 72°; Slice Thickness: 2 mm;  Volumes: 427 |
| Language Assessments | Western Aphasia Battery (WAB-AQ)  Aphasia Battery of Chinese (ABC) | Western Aphasia Battery (WAB-AQ) |
| Covariates | Age, sex, education level, lesion volume, disease duration | Age, sex, lesion volume, disease duration |
| Data Analysis Software | DPARSF; SPM12; MATLAB; MDTB parcellation, connectivity scripts | |
| Preprocessing Pipeline | Discard first 10 volumes; Slice timing correction; Realignment; Co-registration to T1; Normalization (DARTEL);  Nuisance regression (GSR, 24 motion params, WM, CSF); Smooth (4mm FWHM); Filter (0.01-0.1 Hz) | |

Abbreviation: FA = fluent aphasia group; nonFA = non-fluent aphasia group; HC = healthy controls; MDTB = Multi-Domain Task Battery; ARC = Aphasia Recovery Cohort; WAB-AQ = Western Aphasia Battery-Aphasia Quotient; ABC = Aphasia Battery of Chinese; CSF = cerebrospinal fluid; FWHM = full width at half maximum; GSR = global signal regression; TE = echo time; TI = inversion time; TR = repetition time; WM = white matter.

**Supplementary Table S2. The Montreal Neurological Institute (MNI) coordinates for all individual patients in two cohorts.**

|  |  | **Cluster (peak MNI: x, y, z)** | | | | | | |
| --- | --- | --- | --- | --- | --- | --- | --- | --- |
|  |  | **MDTB3** | | **MDTB7** | | **MDTB8** | | **MDTB9** |
|  |  | **Left** | **Right** | **Left** | **Right** | **Left** | **Right** | **Right** |
| FA | Sub1001 | -36, -39, -30 | 18, -39, -48 | -24, -81, -39 | 18, -78, -33 | -27, -81, -39 | 24, -78, -36 | 27, -81, -45 |
|  | Sub1005 | -36, -39, -30 | 18, -39, -48 | -24, -81, -39 | 18, -78, -33 | -27, -81, -39 | 24, -78, -36 | 27, -81, -45 |
|  | Sub1007 | -36, -39, -30 | 18, -39, -48 | -24, -81, -39 | 18, -78, -33 | -27, -81, -39 | 24, -78, -36 | 27, -84, -45 |
|  | Sub1011 | -36, -39, -30 | 18, -39, -48 | -24, -81, -39 | 18, -78, -33 | -27, -81, -39 | 24, -78, -39 | 27, -84, -45 |
|  | Sub1016 | -36, -39, -30 | 18, -39, -48 | -24, -81, -39 | 18, -78, -33 | -27, -81, -39 | 24, -78, -39 | 27, -84, -45 |
|  | Sub1021 | -36, -39, -30 | 18, -39, -48 | -24, -81, -39 | 18, -78, -33 | -27, -81, -39 | 24, -78, -36 | 27, -84, -45 |
|  | Sub1023 | -36, -39, -30 | 18, -39, -48 | -24, -81, -36 | 18, -78, -33 | -27, -81, -39 | 24, -78, -36 | 27, -84, -45 |
|  | Sub1026 | -36, -39, -30 | 18, -39, -48 | -24, -81, -39 | 18, -78, -33 | -27, -81, -39 | 24, -78, -36 | 27, -84, -45 |
|  | Sub1032 | -36, -39, -30 | 18, -39, -48 | -24, -81, -39 | 18, -78, -33 | -27, -81, -39 | 24, -78, -36 | 27, -84, -45 |
|  | Sub1033 | -36, -39, -30 | 18, -39, -48 | -24, -81, -39 | 18, -78, -33 | -27, -81, -39 | 24, -78, -36 | 27, -84, -45 |
|  | Sub1036 | -36, -39, -30 | 18, -39, -48 | -24, -81, -39 | 18, -78, -33 | -27, -81, -39 | 24, -78, -36 | 27, -84, -45 |
|  | Sub1037 | -36, -39, -30 | 18, -39, -48 | -24, -81, -39 | 18, -78, -33 | -27, -81, -39 | 24, -78, -39 | 27, -84, -45 |
|  | Sub1042 | -36, -39, -30 | 18, -39, -48 | -24, -81, -39 | 18, -78, -33 | -27, -81, -39 | 24, -78, -36 | 27, -84, -45 |
|  | Sub1043 | -36, -39, -30 | 18, -39, -48 | -24, -81, -39 | 18, -78, -33 | -27, -81, -39 | 24, -78, -36 | 27, -84, -45 |
|  | Sub1044 | -36, -39, -30 | 18, -39, -48 | -24, -81, -36 | 18, -78, -33 | -27, -81, -39 | 24, -78, -36 | 27, -84, -45 |
|  | Sub1045 | -36, -39, -30 | 18, -39, -48 | -24, -81, -39 | 18, -78, -33 | -27, -81, -39 | 24, -78, -36 | 27, -81, -45 |
|  | Sub1048 | -36, -39, -30 | 18, -39, -48 | -24, -81, -39 | 18, -78, -33 | -27, -81, -39 | 24, -78, -39 | 27, -84, -45 |
|  | Sub1050 | -36, -39, -30 | 18, -39, -48 | -24, -81, -39 | 18, -78, -33 | -27, -81, -39 | 24, -78, -39 | 27, -84, -45 |
|  | Sub1053 | -36, -39, -30 | 18, -39, -48 | -24, -81, -39 | 18, -78, -33 | -27, -81, -39 | 24, -78, -39 | 27, -84, -45 |
|  | Sub1065 | -36, -39, -30 | 18, -39, -48 | -24, -81, -39 | 18, -78, -33 | -27, -81, -39 | 24, -78, -36 | 27, -84, -45 |
|  | Sub1066 | -36, -39, -30 | 18, -39, -48 | -24, -81, -39 | 18, -78, -33 | -27, -81, -39 | 24, -78, -36 | 27, -84, -45 |
|  | Sub1067 | -36, -39, -30 | 18, -39, -48 | -24, -81, -39 | 18, -78, -33 | -27, -81, -39 | 24, -78, -36 | 27, -84, -45 |
|  | Sub1074 | -36, -39, -30 | 18, -39, -48 | -24, -81, -39 | 18, -78, -33 | -27, -81, -39 | 24, -78, -36 | 27, -84, -45 |
|  | Sub1077 | -36, -39, -30 | 18, -39, -48 | -24, -81, -39 | 18, -78, -33 | -27, -81, -39 | 24, -78, -36 | 27, -84, -45 |
|  | Sub1078 | -36, -39, -30 | 18, -39, -48 | -24, -81, -39 | 18, -78, -33 | -27, -81, -39 | 24, -78, -36 | 27, -84, -45 |
|  | Sub1079 | -36, -39, -30 | 18, -39, -48 | -24, -81, -39 | 18, -78, -33 | -27, -81, -39 | 24, -78, -39 | 27, -84, -45 |
|  | Sub1085 | -36, -39, -30 | 18, -39, -48 | -24, -81, -39 | 18, -78, -33 | -27, -81, -39 | 24, -78, -36 | 27, -84, -45 |
|  | Sub1086 | -36, -39, -30 | 18, -39, -48 | -24, -81, -36 | 18, -78, -33 | -27, -81, -39 | 24, -78, -36 | 27, -84, -45 |
|  | Sub1090 | -36, -39, -30 | 18, -39, -48 | -24, -81, -39 | 18, -78, -33 | -27, -81, -39 | 24, -78, -36 | 27, -84, -45 |
|  | Sub1092 | -36, -39, -30 | 18, -39, -48 | -24, -81, -39 | 18, -78, -33 | -27, -81, -39 | 24, -78, -39 | 27, -84, -45 |
|  | Sub1096 | -36, -39, -30 | 18, -39, -48 | -24, -81, -39 | 18, -78, -33 | -27, -81, -39 | 24, -78, -36 | 27, -84, -45 |
|  | Sub1104 | -36, -39, -30 | 18, -39, -48 | -24, -81, -39 | 18, -78, -33 | -27, -81, -39 | 24, -78, -36 | 27, -84, -45 |
|  | Sub1107 | -36, -39, -30 | 18, -39, -48 | -24, -81, -39 | 18, -78, -33 | -27, -81, -39 | 24, -78, -36 | 27, -84, -45 |
|  | Sub1111 | -36, -39, -30 | 18, -39, -48 | -24, -81, -36 | 18, -78, -33 | -27, -81, -39 | 24, -78, -36 | 27, -84, -45 |
|  | Sub1120 | -36, -39, -30 | 18, -39, -48 | -24, -81, -39 | 18, -78, -33 | -27, -81, -39 | 24, -78, -36 | 27, -84, -45 |
| nonFA | Sub1002 | -36, -39, -30 | 18, -39, -48 | -24, -81, -39 | 18, -78, -33 | -27, -81, -39 | 24, -78, -39 | 27, -84, -45 |
|  | Sub1003 | -36, -39, -30 | 18, -39, -48 | -24, -81, -39 | 18, -78, -33 | -27, -81, -39 | 24, -78, -36 | 27, -84, -45 |
|  | Sub1006 | -36, -39, -30 | 18, -39, -48 | -24, -81, -39 | 18, -78, -33 | -27, -81, -39 | 24, -78, -39 | 27, -84, -45 |
|  | Sub1008 | -36, -39, -30 | 18, -39, -48 | -24, -81, -39 | 18, -78, -33 | -27, -81, -39 | 24, -78, -36 | 27, -84, -45 |
|  | Sub1014 | -36, -39, -30 | 18, -39, -48 | -24, -81, -39 | 18, -78, -33 | -27, -81, -39 | 24, -78, -39 | 27, -84, -45 |
|  | Sub1015 | -36, -39, -30 | 18, -39, -48 | -24, -81, -39 | 18, -78, -33 | -27, -81, -39 | 24, -78, -36 | 27, -84, -45 |
|  | Sub1018 | -36, -39, -30 | 18, -39, -48 | -24, -81, -39 | 18, -78, -33 | -27, -81, -39 | 24, -78, -39 | 27, -84, -45 |
|  | Sub1020 | -36, -39, -30 | 18, -39, -48 | -24, -81, -39 | 18, -78, -33 | -27, -81, -39 | 24, -78, -36 | 27, -84, -45 |
|  | Sub1024 | -36, -39, -30 | 18, -39, -48 | -24, -81, -39 | 18, -78, -33 | -27, -81, -39 | 24, -78, -36 | 27, -84, -45 |
|  | Sub1025 | -36, -39, -30 | 18, -39, -48 | -24, -81, -39 | 18, -78, -33 | -27, -81, -39 | 24, -78, -39 | 27, -84, -45 |
|  | Sub1031 | -36, -39, -30 | 18, -39, -48 | -24, -81, -39 | 18, -78, -33 | -27, -81, -39 | 24, -78, -36 | 27, -84, -45 |
|  | Sub1041 | -36, -39, -30 | 18, -39, -48 | -24, -81, -39 | 18, -78, -33 | -27, -81, -39 | 24, -78, -36 | 27, -84, -45 |
|  | Sub1057 | -36, -39, -30 | 18, -39, -48 | -24, -81, -39 | 18, -78, -33 | -27, -81, -39 | 24, -78, -36 | 27, -84, -45 |
|  | Sub1058 | -36, -39, -30 | 18, -39, -48 | -24, -81, -39 | 18, -78, -33 | -27, -81, -39 | 24, -78, -36 | 27, -84, -45 |
|  | Sub1059 | -36, -39, -30 | 18, -39, -48 | -24, -81, -39 | 18, -78, -33 | -27, -81, -39 | 24, -78, -39 | 27, -84, -45 |
|  | Sub1061 | -36, -39, -30 | 18, -39, -48 | -24, -81, -39 | 18, -78, -33 | -27, -81, -39 | 24, -78, -36 | 27, -84, -45 |
|  | Sub1068 | -36, -39, -30 | 18, -39, -48 | -24, -81, -39 | 18, -78, -33 | -27, -81, -39 | 24, -78, -36 | 27, -84, -45 |
|  | Sub1070 | -36, -39, -30 | 18, -39, -48 | -24, -81, -39 | 18, -78, -33 | -27, -81, -39 | 24, -78, -39 | 27, -84, -45 |
|  | Sub1072 | -36, -39, -30 | 18, -39, -48 | -24, -81, -39 | 18, -78, -33 | -27, -81, -39 | 24, -78, -36 | 27, -84, -45 |
|  | Sub1073 | -36, -39, -30 | 18, -39, -48 | -24, -81, -39 | 18, -78, -33 | -27, -81, -39 | 24, -78, -36 | 27, -84, -45 |
|  | Sub1075 | -36, -39, -30 | 18, -39, -48 | -24, -81, -36 | 18, -78, -33 | -27, -81, -39 | 24, -78, -36 | 27, -84, -45 |
|  | Sub1080 | -36, -39, -30 | 18, -39, -48 | -24, -81, -39 | 18, -78, -33 | -27, -81, -39 | 24, -78, -36 | 27, -84, -45 |
|  | Sub1087 | -36, -39, -30 | 18, -39, -48 | -24, -81, -39 | 18, -78, -33 | -27, -81, -39 | 24, -78, -36 | 27, -84, -45 |
|  | Sub1089 | -36, -39, -30 | 18, -39, -48 | -24, -81, -39 | 18, -78, -33 | -27, -81, -39 | 24, -78, -36 | 27, -84, -45 |
|  | Sub1093 | -36, -39, -30 | 18, -39, -48 | -24, -81, -39 | 18, -78, -33 | -27, -81, -39 | 24, -78, -36 | 27, -84, -45 |
|  | Sub1094 | -36, -39, -30 | 18, -39, -48 | -24, -81, -36 | 18, -78, -33 | -27, -81, -39 | 24, -78, -36 | 27, -84, -45 |
|  | Sub1095 | -36, -39, -30 | 18, -39, -48 | -24, -81, -39 | 18, -78, -33 | -27, -81, -39 | 24, -78, -36 | 27, -84, -45 |
|  | Sub1097 | -36, -39, -30 | 18, -39, -48 | -24, -81, -39 | 18, -78, -33 | -27, -81, -39 | 24, -78, -36 | 27, -84, -45 |
|  | Sub1100 | -36, -39, -30 | 18, -39, -48 | -24, -81, -39 | 18, -78, -33 | -27, -81, -39 | 24, -78, -36 | 27, -84, -45 |
|  | Sub1101 | -36, -39, -30 | 18, -39, -48 | -24, -81, -39 | 18, -78, -33 | -27, -81, -39 | 24, -78, -36 | 27, -84, -45 |
|  | Sub1102 | -36, -39, -30 | 18, -39, -48 | -24, -81, -39 | 18, -78, -33 | -27, -81, -39 | 24, -78, -36 | 27, -81, -45 |
|  | Sub1109 | -36, -39, -30 | 18, -39, -48 | -24, -81, -36 | 18, -78, -33 | -27, -81, -39 | 24, -78, -36 | 27, -84, -45 |
|  | Sub1110 | -36, -39, -30 | 18, -39, -48 | -24, -81, -39 | 18, -78, -33 | -27, -81, -39 | 24, -78, -36 | 27, -84, -45 |
|  | Sub1112 | -36, -39, -30 | 18, -39, -48 | -24, -81, -39 | 18, -78, -33 | -27, -81, -39 | 24, -78, -36 | 27, -84, -45 |
|  | Sub1113 | -36, -39, -30 | 18, -39, -48 | -24, -81, -39 | 18, -78, -33 | -27, -81, -39 | 24, -78, -36 | 27, -81, -45 |
|  | Sub1114 | -36, -39, -30 | 18, -39, -48 | -24, -81, -39 | 18, -78, -33 | -27, -81, -39 | 24, -78, -36 | 27, -84, -45 |
|  | Sub1115 | -36, -39, -30 | 18, -39, -48 | -24, -81, -36 | 18, -78, -33 | -27, -81, -39 | 24, -78, -36 | 27, -84, -45 |
|  | Sub1116 | -36, -39, -30 | 18, -39, -48 | -24, -81, -39 | 18, -78, -33 | -27, -81, -39 | 24, -78, -36 | 27, -84, -45 |
|  | Sub1117 | -36, -39, -30 | 18, -39, -48 | -24, -81, -39 | 18, -78, -33 | -27, -81, -39 | 24, -78, -36 | 27, -84, -45 |
|  | Sub1119 | -36, -39, -30 | 18, -39, -48 | -24, -81, -39 | 18, -78, -33 | -27, -81, -39 | 24, -78, -36 | 27, -84, -45 |
|  | Sub1121 | -36, -39, -30 | 18, -39, -48 | -24, -81, -39 | 18, -78, -33 | -27, -81, -39 | 24, -78, -36 | 27, -84, -45 |
|  | Sub1122 | -36, -39, -30 | 18, -39, -48 | -24, -81, -39 | 18, -78, -33 | -27, -81, -39 | 24, -78, -36 | 27, -84, -45 |
|  | Sub1123 | -36, -39, -30 | 18, -39, -48 | -24, -81, -39 | 18, -78, -33 | -27, -81, -39 | 24, -78, -39 | 27, -84, -45 |
|  | Sub1124 | -36, -39, -30 | 18, -39, -48 | -24, -81, -39 | 18, -78, -33 | -27, -81, -39 | 24, -78, -36 | 27, -84, -45 |
|  | Sub1125 | -36, -39, -30 | 18, -39, -48 | -24, -81, -39 | 18, -78, -33 | -27, -81, -39 | 24, -78, -36 | 27, -84, -45 |
|  | Sub1126 | -36, -39, -30 | 18, -39, -48 | -24, -81, -39 | 18, -78, -33 | -27, -81, -39 | 24, -78, -36 | 27, -84, -45 |

Abbreviation: FA = fluent aphasia group; nonFA = non-fluent aphasia group; MNI = The Montreal Neurological Institute; MDTB = Multi-Domain Task Battery.

**Supplementary Table S3. Covariate plan for all primary and sensitivity analyses.**

| Analysis Type | Age | Sex | Education | Lesion Volume | Disease Duration | Preserved LN volume | TIV |
| --- | --- | --- | --- | --- | --- | --- | --- |
| ANCOVA |  |  |  |  |  |  |  |
| Between HC, FA and nonFA | √ | √ | √ |  |  |  |  |
| Between FA and nonFA | √ | √ | √ | √ | √ |  |  |
| Partial Correlation Analysis | √ | √ | √ | √ | √ |  |  |
| Mediation Analysis | √ | √ | √ | √ | √ |  |  |
| Sensitivity Analyses |  |  |  |  |  |  |  |
| Analysis in Matched Subsample | √ | √ | √ | √ | √ |  |  |
| Strict Motion Control (FD < 0.2mm) | √ | √ | √ | √ | √ |  |  |
| Without Global Signal Regression | √ | √ | √ | √ | √ |  |  |
| Controlling for Intracranial Volume | √ | √ | √ | √ | √ |  | √ |
| Controlling for Preserved LN Volume | √ | √ | √ |  | √ | √ |  |

Note: A checkmark (√) indicates that a variable was included as a covariate in the corresponding analysis. The “Sensitivity Analyses” section outlines distinct models performed to validate the robustness of our primary findings under different analytical conditions.

Abbreviations: ANCOVA = Analysis of covariance; LN = classical language network; HC = healthy controls; FA = fluent aphasia group; nonFA = non-fluent aphasia group; FD = framewise displacement; GSR = global signal regression; TIV = total intracranial volume.

**Supplementary Table S4. Comparison of FC and Crus II volume between the HC and PSA subgroups.**

| group | HC (n = 77) | PSA (n = 81) | | ANCOVA | | | | | |
| --- | --- | --- | --- | --- | --- | --- | --- | --- | --- |
|  |  | FA (n = 35) | nonFA (n = 46) | Between HC, FA and nonFA ^a^ | | | Between FA and nonFA ^b^ | | |
|  |  |  |  | *p* value | *F* value | Partial *η^2^* | *p* value | *F* value | Partial *η^2^* |
| **Functional connectivity** |  |  |  |  |  |  |  |  |  |
| MDTB3-LN FC | -0.08 ± 0.13 | -0.04 ± 0.06 | -0.06 ± 0.08 | 0.213 | 1.56 | 0.02 | 0.378 | 0.79 | 0.01 |
| MDTB7-LN FC | -0.01 ± 0.18 | -0.01 ± 0.10 | -0.05 ± 0.09 | 0.272 | 1.31 | 0.02 | 0.084 | 3.07 | 0.04 |
| MDTB8-LN FC | -0.03 ± 0.18 | -0.03 ± 0.13 | -0.08 ± 0.09 | 0.313 | 1.17 | 0.02 | 0.177 | 1.86 | 0.02 |
| MDTB9-LN FC | -0.03 ± 0.15 | -0.05 ± 0.11 | -0.08 ± 0.08 | **0.055** | **2.95** | **0.04** | 0.205 | 1.64 | 0.02 |
| L_MDTB3-LN FC | -0.07 ± 0.12 | -0.05 ± 0.09 | -0.04 ± 0.11 | 0.296 | 1.23 | 0.02 | 0.996 | 0.00 | 0.00 |
| R_MDTB3-LN FC | -0.06 ± 0.10 | -0.05 ± 0.09 | -0.08 ± 0.10 | 0.440 | 0.83 | 0.01 | 0.234 | 1.44 | 0.02 |
| L_MDTB7-LN FC | 0.05 ± 0.21 | 0.07 ± 0.16 | 0.01 ± 0.16 | 0.588 | 0.53 | 0.01 | 0.345 | 0.90 | 0.01 |
| R_MDTB7-LN FC | 0.10 ± 0.15 | 0.02 ± 0.16 | -0.05 ± 0.14 | **< 0.001** | **13.27** | **0.15** | 0.105 | 2.70 | 0.04 |
| L_MDTB8-LN FC | 0.04 ± 0.21 | 0.02 ± 0.17 | -0.04 ± 0.16 | 0.185 | 1.71 | 0.02 | 0.587 | 0.30 | 0.00 |
| R_MDTB8-LN FC | 0.19 ± 0.20 | 0.06 ± 0.16 | -0.04 ± 0.12 | **< 0.001** | **22.94** | **0.23** | **0.008** | **7.47** | **0.09** |
| R_MDTB9-LN FC | 0.18 ± 0.19 | 0.03 ± 0.17 | -0.08 ± 0.13 | **< 0.001** | **32.80** | **0.30** | **0.016** | **6.07** | **0.08** |
| **Volume** |  |  |  |  |  |  |  |  |  |
| L_Crus II volume | 9.21 ± 0.17 | 9.25 ± 0.15 | 9.16 ± 0.19 | 0.168 | 1.81 | 0.02 | 0.168 | 1.94 | 0.03 |
| R_Crus II volume | 9.58 ± 0.19 | 9.58 ± 0.15 | 9.33 ± 0.20 | **< 0.001** | **25.98** | **0.26** | **< 0.001** | **24.39** | **0.25** |

Note: Continuous variables are presented as the mean ± SD. Bold indicates statistical significance (*p* < 0.05) or a trend toward significance. ^a^ ANCOVA of the HC, FA and nonFA groups, adjusting for age, sex and education level. ^b^ ANCOVA of the FA and nonFA groups, adjusting for age, sex, education level, lesion volume and disease duration.

Abbreviations: ANCOVA = Analysis of covariance; HC = healthy controls; MDTB = Multi-Domain Task Battery; LN = classical language network; FC = functional connectivity; Crus II = cerebellar hemispheric lobule Crus II; FA = fluent aphasia group; nonFA = non-fluent aphasia group.

**Supplementary Table S5. Post hoc analysis of FC and Crus II volume across three groups.**

|  | Group comparison | | *p_bonf_* | Cohen’s *d* |
| --- | --- | --- | --- | --- |
| **Functional connectivity** |  |  |  |  |
| MDTB3-LN FC | nonFA | FA | 0.263 | 0.27 |
|  |  | HC | 0.596 | 0.11 |
|  | FA | HC | 0.079 | 0.37 |
|  |  |  |  |  |
| MDTB7-LN FC | nonFA | FA | 0.178 | 0.31 |
|  |  | HC | 0.140 | 0.28 |
|  | FA | HC | 0.877 | 0.04 |
|  |  |  |  |  |
| MDTB8-LN FC | nonFA | FA | 0.295 | 0.25 |
|  |  | HC | 0.134 | 0.29 |
|  | FA | HC | 0.836 | 0.04 |
|  |  |  |  |  |
| MDTB9-LN FC | nonFA | FA | 0.151 | 0.34 |
|  |  | HC | **0.016** | **0.47** |
|  | FA | HC | 0.537 | 0.13 |
|  |  |  |  |  |
| L_MDTB3-LN FC | nonFA | FA | 0.653 | 0.11 |
|  |  | HC | 0.135 | 0.29 |
|  | FA | HC | 0.377 | 0.18 |
|  |  |  |  |  |
| R_MDTB3-LN FC | nonFA | FA | 0.256 | 0.26 |
|  |  | HC | 0.265 | 0.21 |
|  | FA | HC | 0.801 | 0.05 |
|  |  |  |  |  |
| L_MDTB7-LN FC | nonFA | FA | 0.372 | 0.21 |
|  |  | HC | 0.358 | 0.18 |
|  | FA | HC | 0.873 | 0.03 |
|  |  |  |  |  |
| R_MDTB7-LN FC | nonFA | FA | 0.071 | 0.42 |
|  |  | HC | **< 0.001** | **0.96** |
|  | FA | HC | **0.010** | **0.53** |
|  |  |  |  |  |
| L_MDTB8-LN FC | nonFA | FA | 0.356 | 0.22 |
|  |  | HC | 0.067 | 0.35 |
|  | FA | HC | 0.508 | 0.14 |
|  |  |  |  |  |
| R_MDTB8-LN FC | nonFA | FA | **0.028** | **0.52** |
|  |  | HC | **< 0.001** | **1.26** |
|  | FA | HC | **0.001** | **0.74** |
|  |  |  |  |  |
| R_MDTB9-LN FC | nonFA | FA | **0.010** | **0.62** |
|  |  | HC | **< 0.001** | **1.51** |
|  | FA | HC | **< 0.001** | **0.90** |
| **Volume** |  |  |  |  |
| L_Crus II volume | nonFA | FA | 0.060 | 0.45 |
|  |  | HC | 0.257 | 0.22 |
|  | FA | HC | 0.277 | 0.23 |
|  |  |  |  |  |
| R_Crus II volume | nonFA | FA | **< 0.001** | **1.32** |
|  |  | HC | **< 0.001** | **1.32** |
|  | FA | HC | 0.989 | 0.01 |

Note: ANCOVA model adjusted for age, sex and education level in HC, FA and nonFA; Bold indicates statistical significance (*p_bonf_* < 0.05).

Abbreviations: ANCOVA = Analysis of covariance; HC = healthy controls; MDTB = Multi-Domain Task Battery; LN = classical language network; FC = functional connectivity; Crus II = cerebellar hemispheric lobule Crus II; FA = fluent aphasia group; nonFA = non-fluent aphasia group.

**Supplementary Table S6. Comparison of atlas-based centroids and individualized peak functional connectivity.**

| Predictor Models | ANCOVA | | |  | Hierarchical Regression | | | | | |
| --- | --- | --- | --- | --- | --- | --- | --- | --- | --- | --- |
|  | *F* | *p* | Partial *η^2^* |  | Model *R^2^* | Δ*R^2^* | *F* change | *p* change | AIC | ΔAIC |
| MDTB7 |  |  |  |  |  |  |  |  |  |  |
| Model 1: Atlas-based centroids | 0.07 | 0.800 | 0.00 |  | 0.31 | - | - | - | 534.61 | - |
| Model 2: Individualized | 2.70 | 0.105 | 0.04 |  | 0.39 | +0.08 | 8.94 | 0.004 | 527.20 | +7.41 |
| MDTB8 |  |  |  |  |  |  |  |  |  |  |
| Model 1: Atlas-based centroids | **4.91** | **0.030** | **0.06** |  | 0.30 | - | - | - | 536.01 | - |
| Model 2: Individualized | **7.47** | **0.008** | **0.09** |  | 0.41 | +0.11 | 13.13 | 0.001 | 521.96 | +14.05 |
| MDTB9 |  |  |  |  |  |  |  |  |  |  |
| Model 1: Atlas-based centroids | 2.82 | 0.097 | 0.04 |  | 0.31 | - | - | - | 535.35 | - |
| Model 2: Individualized | **6.07** | **0.016** | **0.08** |  | 0.35 | +0.04 | 4.79 | 0.032 | 533.15 | +2.20 |

Note: ANCOVA reports group comparisons on FC between FA and nonFA groups (HC: n = 77; FA: n = 35; nonFA: n = 46). Hierarchical Regression analysis shows results from models predicting AQ scores. Model 1, the baseline model, includes the atlas-based FC and covariates (age, sex, education level, lesion volume and disease duration). Model 2 expands on Model 1 by adding the individualized peak FC. ΔR^2^ reflects the significant increase in explained variance attributable to the individualized predictor. AIC indicating model fit. ΔAIC represents the change in AIC from Model 1 to Model 2 (AIC_1_ - AIC_2_). Bold indicates statistical significance (*p* < 0.05).

Abbreviations: ANCOVA = Analysis of covariance; HC = healthy controls; MDTB = Multi-Domain Task Battery; LN = classical language network; FC = functional connectivity; AQ = Aphasia Quotient; FA = fluent aphasia group; nonFA = non-fluent aphasia group; AIC = Akaike Information Criterion.

**Supplementary Table S7. Partial correlation analyses were used to assess the relationships between neuroimaging indicators and language performance in patients with nonFA (n = 46).**

| indicators |  | L_CrusII volume | R_CrusII volume | L_MDTB7-LN FC | R_MDTB7-LN FC | L_MDTB8-LN FC | R_MDTB8-LN FC | R_MDTB9-LN FC |
| --- | --- | --- | --- | --- | --- | --- | --- | --- |
| AQ | *r* | 0.20 | 0.68 | 0.361 | 0.25 | 0.19 | 0.56 | 0.41 |
|  | *p* value | 0.219 | **<0.001** | **0.021** | 0.109 | 0.248 | **<0.001** | **0.007** |
| Fluency | *r* | 0.06 | 0.75 | 0.32 | 0.43 | 0.17 | 0.52 | 0.30 |
|  | *p* value | 0.719 | **<0.001** | **0.044** | **0.005** | 0.281 | **0.001** | **0.057** |
| Information | *r* | 0.18 | 0.72 | 0.33 | 0.19 | 0.23 | 0.57 | 0.40 |
|  | *p* value | 0.360 | **<0.001** | **0.034** | 0.065 | 0.156 | **<0.001** | **0.010** |
| Spontaneous speech | *r* | 0.12 | 0.77 | 0.34 | 0.36 | 0.21 | 0.58 | 0.38 |
|  | *p* value | 0.466 | **<0.001** | **0.029** | **0.020** | 0.179 | **<0.001** | **0.015** |
| Auditory Comprehension | *r* | 0.23 | 0.41 | 0.45 | 0.07 | 0.10 | 0.38 | 0.25 |
|  | *p* value | 0.145 | **0.008** | **0.003** | 0.662 | 0.525 | **0.016** | 0.121 |
| Repetition | *r* | 0.16 | 0.38 | 0.07 | 0.07 | 0.03 | 0.36 | 0.37 |
|  | *p* value | 0.317 | **0.015** | 0.686 | 0.672 | 0.836 | **0.023** | **0.017** |
| Naming | *r* | 0.15 | 0.57 | 0.25 | 0.26 | 0.22 | 0.45 | 0.36 |
|  | *p* value | 0.367 | **<0.001** | 0.122 | 0.108 | 0.174 | **0.003** | **0.022** |

Note: Values represent partial correlation coefficients (*r*) and corresponding *p-*values, adjusted for age, sex, educational level, lesion volume, and disease duration. Bold indicates statistical significance (*p* < 0.05).

Abbreviations: AQ = Aphasia Quotient; MDTB = Multi-Domain Task Battery; LN = classical language network; FC = functional connectivity; Crus II = cerebellar hemispheric lobule Crus II; FA = fluent aphasia group; nonFA = non-fluent aphasia group.

**Supplementary Table S8. Partial correlation analyses were used to assess the relationships between neuroimaging indicators and language performance in patients with FA (n = 35).**

| indicators |  | L_CrusII volume | R_CrusII volume | L_MDTB7-LN FC | R_MDTB7-LN FC | L_MDTB8-LN FC | R_MDTB8-LN FC | R_MDTB9-LN FC |
| --- | --- | --- | --- | --- | --- | --- | --- | --- |
| AQ | *r* | 0.13 | -0.02 | -0.04 | 0.15 | -0.17 | -0.02 | -0.18 |
|  | *p* value | 0.487 | 0.938 | 0.841 | 0.421 | 0.369 | 0.900 | 0.351 |
| Fluency | *r* | 0.29 | -0.05 | -0.23 | -0.03 | -0.331 | -0.17 | -0.27 |
|  | *p* value | 0.127 | 0.798 | 0.231 | 0.867 | 0.074 | 0.367 | 0.148 |
| Information | *r* | 0.09 | 0.01 | 0.02 | 0.18 | -0.06 | 0.11 | -0.09 |
|  | *p* value | 0.653 | 0.970 | 0.920 | 0.347 | 0.736 | 0.562 | 0.648 |
| Spontaneous speech | *r* | 0.19 | -0.02 | -0.09 | 0.10 | -0.19 | -0.01 | -0.18 |
|  | *p* value | 0.326 | 0.926 | 0.634 | 0.598 | 0.307 | 0.972 | 0.342 |
| Auditory Comprehension | *r* | 0.03 | -0.04 | 0.15 | 0.20 | 0.06 | 0.18 | 0.04 |
|  | *p* value | 0.868 | 0.835 | 0.424 | 0.122 | 0.741 | 0.347 | 0.850 |
| Repetition | *r* | 0.01 | -0.11 | -0.13 | 0.07 | -0.26 | -0.17 | -0.19 |
|  | *p* value | 0.974 | 0.573 | 0.504 | 0.724 | 0.164 | 0.366 | 0.317 |
| Naming | *r* | 0.18 | 0.10 | 0.001 | 0.12 | -0.12 | -0.02 | -0.20 |
|  | *p* value | 0.333 | 0.610 | 0.999 | 0.524 | 0.524 | 0.901 | 0.281 |

Note: Values represent partial correlation coefficients (*r*) and corresponding *p-*values, adjusted for age, sex, educational level, lesion volume, and disease duration. Bold indicates statistical significance (*p* < 0.05).

Abbreviations: AQ = Aphasia Quotient; MDTB = Multi-Domain Task Battery; LN = classical language network; FC = functional connectivity; Crus II = cerebellar hemispheric lobule Crus II; FA = fluent aphasia group; nonFA = non-fluent aphasia group.

**Supplementary Table S9. Prerequisite regression (path c', X→Y): hierarchical analysis of R_MDTB8-LN FC on AQ scores adjusted for covariates.**

| **Variables** | **Model 1**  R_Crus II volume | | **Model 2**  R_MDTB8-LN FC | | **Model 3**  R_Crus II volume | |
| --- | --- | --- | --- | --- | --- | --- |
|  | *β* | *t* | *β* | *t* | *β* | *t* |
| Age | -0.12 | -0.81 | -0.06 | -0.44 | -0.01 | -0.01 |
| Education | 0.08 | 0.48 | 0.36 | 2.28* | 0.32 | 2.36* |
| Sex | -0.02 | -0.10 | 0.12 | 0.78 | 0.12 | 0.97 |
| Lesion volume | 0.09 | 0.62 | -0.14 | -1.04 | -0.19 | -1.60 |
| Disease duration | -0.10 | -0.65 | -0.03 | -0.18 | 0.02 | 0.19 |
| R_MDTB8-LN FC | 0.55 | 4.21 *** | 0.52 | 4.19 *** | 0.24 | 1.87 |
| R_Crus II volume | - | - | - | - | 0.51 | 3.91 *** |
| R^2^ | 0.59 | | 0.65 | | 0.76 | |
| △R^2^ | 0.35 | | 0.42 | | 0.58 | |
| F | 3.50 ** | | 4.66 ** | | 7.64 *** | |

Abbreviations: AQ = Aphasia Quotient; MDTB = Multi-Domain Task Battery; LN = classical language network; FC = functional connectivity; Crus II = cerebellar hemispheric lobule Crus II; FA = fluent aphasia group; nonFA = non-fluent aphasia group. *β* = standardized regression coefficient; **p* < 0.05, ** *p* < 0.01, *** *p* < 0.001.

**Supplementary Table S10. Prerequisite regression (path c', X→Y): hierarchical analysis of R_MDTB9-LN FC on AQ scores adjusted for covariates.**

| **Variables** | **Model 1**  R_Crus II volume | | **Model 2**  R_MDTB9-LN FC | | **Model 3**  R_Crus II volume | |
| --- | --- | --- | --- | --- | --- | --- |
|  | *β* | *t* | *β* | *t* | *β* | *t* |
| Age | -0.17 | -1.07 | -0.11 | -0.71 | -0.01 | -0.07 |
| Education | -0.06 | -0.34 | 0.23 | 1.30 | 0.27 | 1.87 |
| Sex | -0.13 | -0.76 | 0.01 | 0.07 | 0.09 | 0.66 |
| Lesion volume | 0.10 | 0.65 | -0.13 | -0.88 | -0.19 | -1.58 |
| Disease duration | 0.01 | 0.02 | 0.06 | 0.39 | 0.06 | 0.47 |
| R_MDTB9-LN FC | 0.46 | 3.04 ** | 0.41 | 2.83 ** | 0.14 | 1.09 |
| R_Crus II volume | - | - | - | - | 0.58 | 4.74 *** |
| R^2^ | 0.49 | | 0.55 | | 0.75 | |
| △R^2^ | 0.24 | | 0.30 | | 0.56 | |
| F | 2.09* | | 2.78* | | 6.91 *** | |

Abbreviations: AQ = Aphasia Quotient; MDTB = Multi-Domain Task Battery; LN = classical language network; FC = functional connectivity; Crus II = cerebellar hemispheric lobule Crus II; FA = fluent aphasia group; nonFA = non-fluent aphasia group. *β* = standardized regression coefficient; **p* < 0.05, ** *p* < 0.01, *** *p* < 0.001.

**Supplementary Table S11. Mediation effect of R_MDTB8-LN FC on AQ scores via R_Crus II volume in nonFA group of the primary cohort (n = 46).**

| Effect Type | Path | *β* | BootSE | Bootstrap 95%CI | | Ratios of Total and Indirect effects (%) |
| --- | --- | --- | --- | --- | --- | --- |
|  |  |  |  | LLCI | ULCI |  |
| Total effect | R_MDTB8-LN FC→AQ | 0.52 | 0.12 | 0.27 | 0.77 | 100.00 |
| Direct effect | R_MDTB8-LN FC→AQ | 0.24 | 0.13 | -0.02 | 0.50 | 46.15 |
| Indirect effect | R_MDTB8-LN FC→R_Crus II volume→AQ | 0.28 | 0.10 | 0.12 | 0.51 | 53.85 |

Note: The mediation model adjusted for age, sex, education level, lesion volume and disease duration. 95% CI based on bootstrapping (5,000 samples).

Abbreviations: SE = standard error; CI = confidence interval; LLCI = lower level of confidence interval; ULCI = upper level of confidence interval; AQ = Aphasia Quotient; MDTB = Multi-Domain Task Battery; LN = classical language network; FC = functional connectivity; Crus II = cerebellar hemispheric lobule Crus II; FA = fluent aphasia group; nonFA = non-fluent aphasia group; *β* = standardized regression coefficient.

**Supplementary Table S12. Mediation effect of R_MDTB9-LN FC on AQ scores via R_Crus II volume in nonFA group of the primary cohort (n = 46)**

| Effect Type | Path | *β* | BootSE | Bootstrap 95%CI | | Ratios of Total and Indirect effects (%) |
| --- | --- | --- | --- | --- | --- | --- |
|  |  |  |  | LLCI | ULCI |  |
| Total effect | R_MDTB9-LN FC→AQ | 0.41 | 0.14 | 0.12 | 0.70 | 100.00 |
| Direct effect | R_MDTB9-LN FC→AQ | 0.14 | 0.13 | -0.12 | 0.40 | 34.15 |
| Indirect effect | R_MDTB9-LN FC→R_Crus II volume→AQ | 0.27 | 0.10 | 0.09 | 0.51 | 65.85 |

Note: The mediation model adjusted for age, sex, education level, lesion volume and disease duration. 95% CI based on bootstrapping (5,000 samples).

Abbreviations: SE = standard error; CI = confidence interval; LLCI = lower level of confidence interval; ULCI = upper level of confidence interval; AQ = Aphasia Quotient; MDTB = Multi-Domain Task Battery; LN = classical language network; FC = functional connectivity; Crus II = cerebellar hemispheric lobule Crus II; FA = fluent aphasia group; nonFA = non-fluent aphasia group; *β* = standardized regression coefficient.

**Supplementary Table S13. Mediation effect of cerebellar volume on AQ scores via functional connectivity in nonFA group of the primary cohort (n = 46).**

|  | Effect Type | Path | *β* | BootSE | Bootstrap 95%CI | | Ratios of Total and Indirect effects (%) |
| --- | --- | --- | --- | --- | --- | --- | --- |
|  |  |  |  |  | LLCI | ULCI |  |
| R_MDTB8-LN FC pathway | Total effect | R_Crus II volume→AQ | 0.56 | 0.08 | 0.40 | 0.72 | 100.00 |
|  | Direct effect | R_MDTB8-LN FC→AQ | 0.50 | 0.09 | 0.32 | 0.69 | 90.61 |
|  | Indirect effect | R_Crus II volume→R_MDTB8-LN FC→AQ | 0.05 | 0.05 | -0.05 | 0.17 | 9.39 |
|  |  |  |  |  |  |  |  |
| R_MDTB9-LN FC pathway | Total effect | R_Crus II volume→AQ | 0.56 | 0.08 | 0.39 | 0.72 | 100.00 |
|  | Direct effect | R_MDTB9-LN FC→AQ | 0.54 | 0.09 | 0.37 | 0.72 | 97.64 |
|  | Indirect effect | R_Crus II volume→R_MDTB9-LN FC→AQ | 0.01 | 0.04 | -0.05 | 0.10 | 2.36 |

Note: This table presents the results of a mediation analysis examining the mediating role of FC in the relationship between right Crus II volume and AQ scores. The mediation model adjusted for age, sex, education level, lesion volume and disease duration. 95% CI based on bootstrapping (5,000 samples).

Abbreviations: SE = standard error; CI = confidence interval; LLCI = lower level of confidence interval; ULCI = upper level of confidence interval; AQ = Aphasia Quotient; MDTB = Multi-Domain Task Battery; LN = classical language network; FC = functional connectivity; Crus II = cerebellar hemispheric lobule Crus II; FA = fluent aphasia group; nonFA = non-fluent aphasia group; *β* = standardized regression coefficient.

**Supplementary Table S14. Sensitivity analysis: Robustness of key findings in a demographically matched subsample.**

**(A) Demographic and clinical characteristics of the matched cohort.**

| group | HC  (n = 75) | PSA (n = 70) | | T-test | | ANOVA |
| --- | --- | --- | --- | --- | --- | --- |
|  |  | FA  (n = 29) | nonFA  (n = 41) | *p* value  (HC and PSA) | *p* value  (FA and nonFA) | *p* value |
| Age (years) | 55.17 ± 11.11 | 55.21 ± 9.36 | 57.73 ± 8.81 | 0.399 ^d^ | 0.254 ^c^ | 0.400 ^a^ |
| Sex (male/female) | 48/27 | 21/8 | 30/11 | 0.334 ^b^ | 0.999 ^b^ | 0.518 ^b^ |
| Education (years) | 9.32 ± 3.75 | 8.97 ± 4.96 | 7.93 ± 4.00 | 0.243 ^d^ | 0.213 ^d^ | 0.215 ^a^ |
| Total intracranial volume (cm^3^) | 1518.74 ± 126.00 | 1552.96 ± 111.74 | 1526.41 ± 128.88 | 0.434 ^d^ | 0.373 ^c^ | 0.452 ^a^ |
| Disease duration (weeks) | - | 28.93 ± 84.69 | 8.98 ± 14.25 | - | 0.627 ^d^ | - |
| Lesion volume (cm^3^) | - | 32.24 ± 31.88 | 47.14 ± 48.55 | - | 0.187 ^d^ | - |
| Preserved LN volume (cm^3^) | - | 23.79 ± 3.70 | 23.33 ± 4.97 | - | 0.988 ^d^ | - |
| **Language scores** |  |  |  |  |  |  |
| Fluency ^e^ | - | 7.41 ± 1.64 | 1.80 ± 1.82 | - | **<0.001** ^d^ | - |
| AQ scores ^e^ | - | 67.01 ± 22.75 | 28.10 ± 22.60 | - | **<0.001** ^d^ | - |

**(B) Comparison of ANCOVA results before and after demographic matching.**

|  |  | ANCOVA | | | | | | | |
| --- | --- | --- | --- | --- | --- | --- | --- | --- | --- |
|  | Analysis Model | Between HC, FA and nonFA ^a^ | | |  | Between FA and nonFA ^b^ | | |  |
|  |  | *p-*value | *F*-statistic | Partial *η^2^* |  | *p-*value | *F*-statistic | Partial *η^2^* |  |
| MDTB9-LN FC | Primary Analysis | **0.055** | **2.95** | **0.04** |  | 0.205 | 1.64 | 0.02 |  |
|  | Analysis in Matched Subsample | 0.129 | 2.08 | 0.03 |  | 0.398 | 0.72 | 0.01 |  |
| R_MDTB7-LN FC | Primary Analysis | **< 0.001** | **13.27** | **0.15** |  | 0.105 | 2.70 | 0.04 |  |
|  | Analysis in Matched Subsample | **< 0.001** | **12.10** | **0.15** |  | 0.119 | 2.49 | 0.04 |  |
| R_MDTB8-LN FC | Primary Analysis | **< 0.001** | **22.94** | **0.23** |  | **0.008** | **7.47** | **0.09** |  |
|  | Analysis in Matched Subsample | **< 0.001** | **21.22** | **0.23** |  | **0.011** | **6.92** | **0.10** |  |
| R_MDTB9-LN FC | Primary Analysis | **< 0.001** | **32.80** | **0.30** |  | **0.016** | **6.07** | **0.08** |  |
|  | Analysis in Matched Subsample | **< 0.001** | **31.46** | **0.31** |  | **0.034** | **4.67** | **0.07** |  |
| R_Crus II volume | Primary Analysis | **< 0.001** | **25.98** | **0.26** |  | **< 0.001** | **24.39** | **0.25** |  |
|  | Analysis in Matched Subsample | **< 0.001** | **22.84** | **0.25** |  | **< 0.001** | **21.20** | **0.25** |  |

Note: This table presents the sensitivity analysis performed on a demographically matched subsample (HC: n = 75; FA: n = 29; nonFA: n = 41). Bold indicates statistical significance (*p* < 0.05) or a trend toward significance.
(A) Continuous variables are presented as the means ± SD, and categorical variables are presented as counts (n). Bold indicates statistical significance (*p* < 0.05). ^a^ ANOVA between HC, FA and nonFA groups. ^b^ Chi-squared test. ^c^ independent samples T-test between FA and nonFA. ^d^ Mann-Whitney U test. ^e^ standardized scores according to Western Aphasia Battery.

(B) ^a^ ANCOVA of the HC, FA and nonFA groups, adjusting for age, sex and education level. ^b^ ANCOVA of the FA and nonFA groups, adjusting for age, sex, education level, lesion volume and disease duration.

Abbreviations: ANCOVA = Analysis of covariance; HC = healthy controls; MDTB = Multi-Domain Task Battery; LN = classical language network; FC = functional connectivity; Crus II = cerebellar hemispheric lobule Crus II; PSA = post stroke aphasia; FA = fluent aphasia group; nonFA = non-fluent aphasia group.

**Supplementary Table S15. Sensitivity analysis: Robustness of key findings after replacing lesion volume with preserved LN volume as a covariate.**

1. **Robustness of group differences.**

|  | Covariate | ANCOVA | | |  |  |  |
| --- | --- | --- | --- | --- | --- | --- | --- |
|  |  | *p-*value | *F*-statistic | Partial *η^2^* |  | F-statistic | Partial *η^2^* |
| MDTB9-LN FC | Lesion Volume | 0.205 | 1.64 | 0.02 |  |  |  |
|  | Preserved LN | 0.180 | 1.84 | 0.02 |  |  |  |
| R_MDTB7-LN FC | Lesion Volume | 0.105 | 2.70 | 0.04 |  |  |  |
|  | Preserved LN | 0.069 | 3.41 | 0.04 |  |  |  |
| R_MDTB8-LN FC | Lesion Volume | **0.008** | **7.47** | **0.09** |  |  |  |
|  | Preserved LN | **0.004** | **8.61** | **0.10** |  |  |  |
| R_MDTB9-LN FC | Lesion Volume | **0.016** | **6.07** | **0.08** |  |  |  |
|  | Preserved LN | **0.009** | **7.14** | **0.09** |  |  |  |
| R_Crus II volume | Lesion Volume | **< 0.001** | **24.39** | **0.25** |  |  |  |
|  | Preserved LN | **< 0.001** | **25.85** | **0.26** |  |  |  |

1. **Robustness of Cerebellar FC and AQ correlations in nonFA group (n = 46).**

|  | Primary Analysis | | Sensitivity Analysis | |
| --- | --- | --- | --- | --- |
|  | *r* [95% CI] | *p_FDR_* | *r* [95% CI] | *p_FDR_* |
| MDTB9-LN FC | -0.08 [-0.38, 0.23] | 0.620 | -0.09 [-0.39, 0.22] | 0.576 |
| R_MDTB7-LN FC | 0.25 [-0.06, 0.52] | 0.136 | 0.24 [-0.07, 0.51] | 0.165 |
| R_MDTB8-LN FC | **0.56 [0.30, 0.74]** | **0.001** | **0.53 [0.26, 0.72]** | **0.001** |
| R_MDTB9-LN FC | **0.41 [0.12, 0.64]** | **0.009** | **0.39 [0.09, 0.62]** | **0.022** |
| R_Crus II volume | **0.68 [0.47, 0.82]** | **0.001** | **0.67 [0.46, 0.81]** | **0.001** |

**(C) Mediation analysis using preserved LN volume as a covariate in nonFA group (n = 46).**

|  | Effect Type | Path | *β* | BootSE | Bootstrap 95%CI | | Ratios of Total and Indirect effects (%) |
| --- | --- | --- | --- | --- | --- | --- | --- |
|  |  |  |  |  | LLCI | ULCI |  |
| R_MDTB8-LN FC pathway | Total effect | R_MDTB8-LN FC→AQ | 0.35 | 0.09 | 0.16 | 0.53 | 100.00 |
|  | Direct effect | R_MDTB8-LN FC→AQ | 0.10 | 0.09 | -0.08 | 0.28 | 28.84 |
|  | Indirect effect | R_MDTB8-LN FC→R_Crus II volume→AQ | 0.25 | 0.06 | 0.13 | 0.39 | 71.16 |
|  |  |  |  |  |  |  |  |
| R_MDTB9-LN FC pathway | Total effect | R_MDTB9-LN FC→AQ | 0.21 | 0.10 | 0.01 | 0.42 | 100.00 |
|  | Direct effect | R_MDTB9-LN FC→AQ | 0.02 | 0.09 | -0.15 | 0.20 | 9.70 |
|  | Indirect effect | R_MDTB9-LN FC→R_Crus II volume→AQ | 0.19 | 0.06 | 0.08 | 0.34 | 90.30 |

Note: This table presents the results of a sensitivity analysis designed to confirm the robustness of the primary findings. In all sensitivity models presented here, the original lesion volume covariate was replaced with preserved LN volume (HC: n = 77; FA: n = 35; nonFA: n = 46).

(A) This section compares the results of two ANCOVA models for group differences between the FA and nonFA groups. The first model (Lesion Volume) is the primary analysis, while the second (Preserved LN) is the sensitivity analysis.

(B) This section compares the results of two partial correlation models examining brain-behavior relationships in the nonFA group.

(C) This section presents the results of a mediation analysis conducted within the nonFA group, using the sensitivity model (controlling for age, sex, education level, disease duration, and preserved LN volume). 95% CI based on bootstrapping (5,000 samples).

Abbreviations: *β* = standardized regression coefficient; SE = standard error; CI = confidence interval; LLCI = lower level of confidence interval; ULCI = upper level of confidence interval; AQ = Aphasia Quotient; MDTB = Multi-Domain Task Battery; LN = classical language network; FC = functional connectivity; Crus II = cerebellar hemispheric lobule Crus II; FA = fluent aphasia group; nonFA = non-fluent aphasia group.

**Supplementary Table S16. Sensitivity analysis:** **Robustness of Crus II volume group differences after controlling for TIV.**

|  |  | ANCOVA | | | | | |
| --- | --- | --- | --- | --- | --- | --- | --- |
|  |  | Between HC, FA and nonFA ^a^ | | | Between FA and nonFA ^b^ | | |
|  |  | *p-*value | *F*-statistic | Partial *η^2^* | *p-*value | F-statistic | Partial *η^2^* |
| L_Crus II volume | Primary Analysis | 0.168 | 1.81 | 0.02 | 0.168 | 1.94 | 0.03 |
|  | Controlling TIV | 0.179 | 1.74 | 0.02 | 0.171 | 1.92 | 0.03 |
| R_Crus II volume | Primary Analysis | **< 0.001** | **25.98** | **0.26** | **< 0.001** | **24.39** | **0.25** |
|  | Controlling TIV | **< 0.001** | **25.89** | **0.26** | **< 0.001** | **24.13** | **0.25** |

Notes: This table presents the results of a sensitivity analysis to test the robustness of Crus II volume findings after accounting for individual differences in TIV (HC: n = 77; FA: n = 35; nonFA: n = 46). The Primary Analysis refers to the original ANCOVA models. The Controlling TIV analysis refers to the sensitivity models, which included all covariates from the primary models plus TIV as an additional covariate. ^a^ ANCOVA of the HC, FA and nonFA groups, adjusting for age, sex, education level and TIV. ^b^ ANCOVA of the FA and nonFA groups, adjusting for age, sex, education level, lesion volume, disease duration and TIV. Bold indicates statistical significance (*p* < 0.05).

Abbreviations: ANCOVA = Analysis of covariance; TIV = total intracranial volume; Crus II = cerebellar hemispheric lobule Crus II; FA = fluent aphasia group; nonFA = non-fluent aphasia group; HC =healthy controls.

**Supplementary Table S17. Sensitivity analysis: Robustness of ANCOVA key findings to stricter head motion control and global signal regression.**

|  |  | ANCOVA | | | | | |
| --- | --- | --- | --- | --- | --- | --- | --- |
|  |  | Between HC, FA and nonFA ^a^ | | | Between FA and nonFA ^b^ | | |
|  |  | *p-*value | *F*-statistic | Partial *η^2^* | *p-*value | F-statistic | Partial *η^2^* |
| MDTB9-LN FC | Primary Analysis | **0.055** | **2.95** | **0.04** | 0.205 | 1.64 | 0.02 |
|  | Sensitivity (FD < 0.2): with GSR | 0.070 | 2.71 | 0.04 | 0.161 | 2.01 | 0.03 |
|  | Sensitivity (FD < 0.2): without GSR | **0.004** | **5.88** | **0.08** | 0.277 | 1.20 | 0.02 |
| R_MDTB7-LN FC | Primary Analysis | **< 0.001** | **13.27** | **0.15** | 0.105 | 2.70 | 0.04 |
|  | Sensitivity (FD < 0.2): with GSR | **< 0.001** | **12.62** | **0.16** | **0.016** | **6.18** | **0.10** |
|  | Sensitivity (FD < 0.2): without GSR | **< 0.001** | **8.86** | **0.12** | 0.154 | 2.09 | 0.04 |
| R_MDTB8-LN FC | Primary Analysis | **< 0.001** | **22.94** | **0.23** | **0.008** | **7.47** | **0.09** |
|  | Sensitivity (FD < 0.2): with GSR | **< 0.001** | **22.00** | **0.25** | **0.006** | **8.14** | **0.12** |
|  | Sensitivity (FD < 0.2): without GSR | **< 0.001** | **18.12** | **0.22** | **0.027** | **5.16** | **0.08** |
| R_MDTB9-LN FC | Primary Analysis | **< 0.001** | **32.80** | **0.30** | **0.016** | **6.07** | **0.08** |
|  | Sensitivity (FD < 0.2): with GSR | **< 0.001** | **27.16** | **0.29** | **0.037** | **4.56** | **0.07** |
|  | Sensitivity (FD < 0.2): without GSR | **< 0.001** | **13.93** | **0.18** | **0.044** | **4.25** | **0.07** |

Note: This table presents the sensitivity analyses testing the robustness of our key findings under two conditions: (1) restricting the analyses to participants with low head motion (mean FD < 0.2 mm), and (2) omitting global signal regression (GSR) in this low-motion subsample (HC: n = 72; FA: n = 25; nonFA: n = 40). Bold indicates statistical significance (*p* < 0.05) or a trend toward significance. ^a^ ANCOVA of the HC, FA and nonFA groups, adjusting for age, sex and education level. ^b^ ANCOVA of the FA and nonFA groups, adjusting for age, sex, education level, lesion volume and disease duration.

Abbreviations: ANCOVA = Analysis of covariance; HC = healthy controls; MDTB = Multi-Domain Task Battery; LN = classical language network; FC = functional connectivity; FA = fluent aphasia group; nonFA = non-fluent aphasia group; AQ = aphasia quotient. FD = Framewise Displacement; GSR = global signal regression.

**Supplemental Figures**


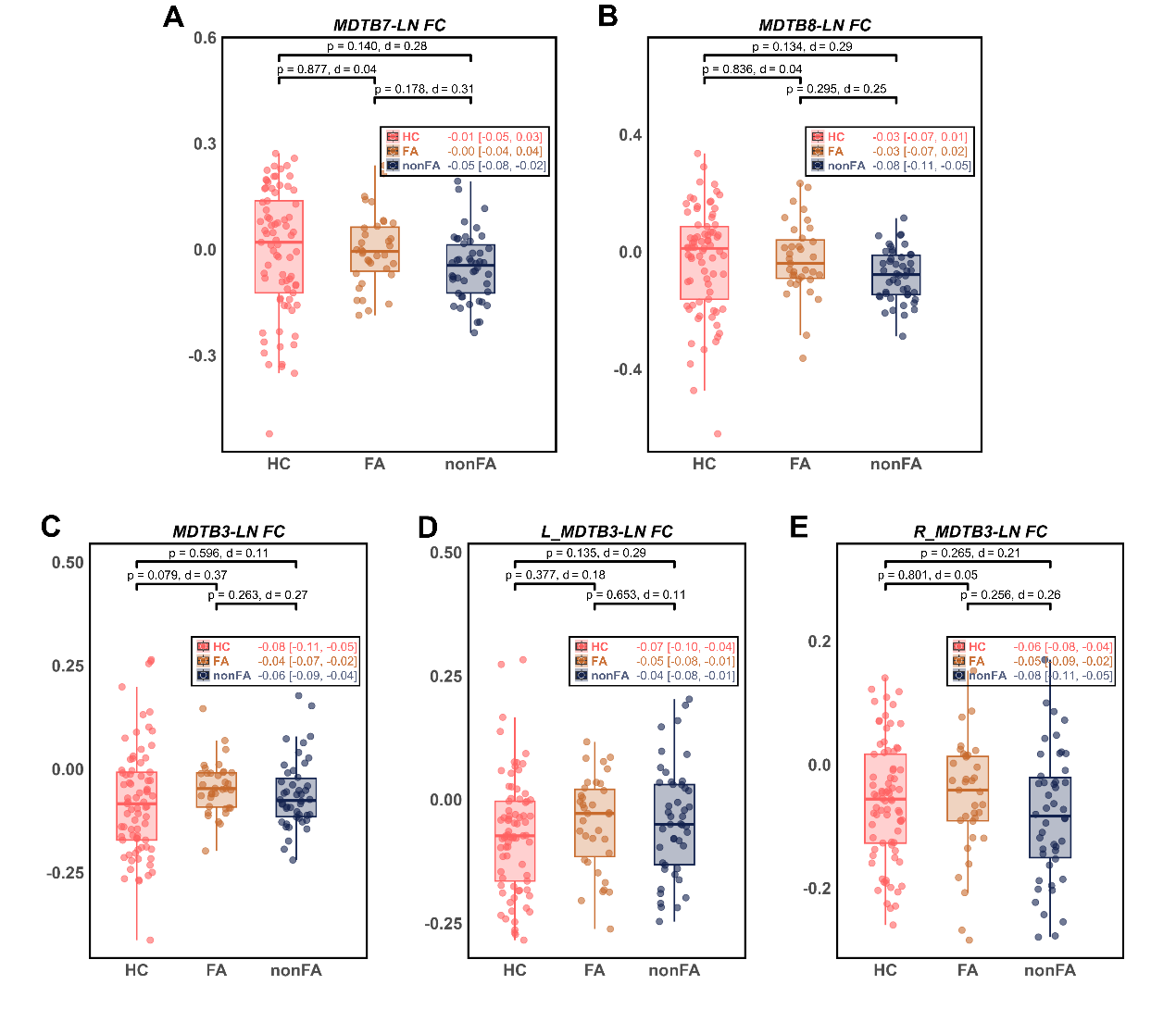


**Supplementary Figure S1 Group differences in functional connectivity using ANCOVA in primary cohort.**

Group comparison of (A) MDTB7-LN FC (*F_(2,152)_* = 1.31, *p* = 0.272, *η^2^* = 0.02), (B) MDTB8-LN FC (*F_(2,152)_* = 1.17, *p* = 0.313, *η^2^* = 0.02), (C) MDTB3-LN FC (*F_(2,152)_* = 1.56, *p* = 0.213, *η^2^* = 0.02), (D) L_MDTB3-LN FC (*F_(2,152)_* = 1.23, *p* = 0.296, *η^2^* = 0.02) and (E) R_MDTB3-LN FC (*F_(2,152)_* = 0.83, *p* = 0.440, *η^2^* = 0.01), adjusted for age, sex and education level. Post-hoc pairwise comparisons utilized Bonferroni-corrected. Bold font indicates statistical significance (*p* < 0.05, Bonferroni-corrected). Box plots display medians (central lines) and IQRs (box boundaries), with overlaid scatter points showing individual data. Inset labels provide group means and 95% CIs. Brackets indicate post-hoc *p*-values and Cohen’s *d*.

Abbreviations: ANCOVA = Analysis of covariance; AQ = Aphasia Quotient; MDTB = Multi-Domain Task Battery; LN = classical language network; FC = functional connectivity; FA = fluent aphasia group; nonFA = non-fluent aphasia group; IQR = interquartile range; CI = confidence interval.


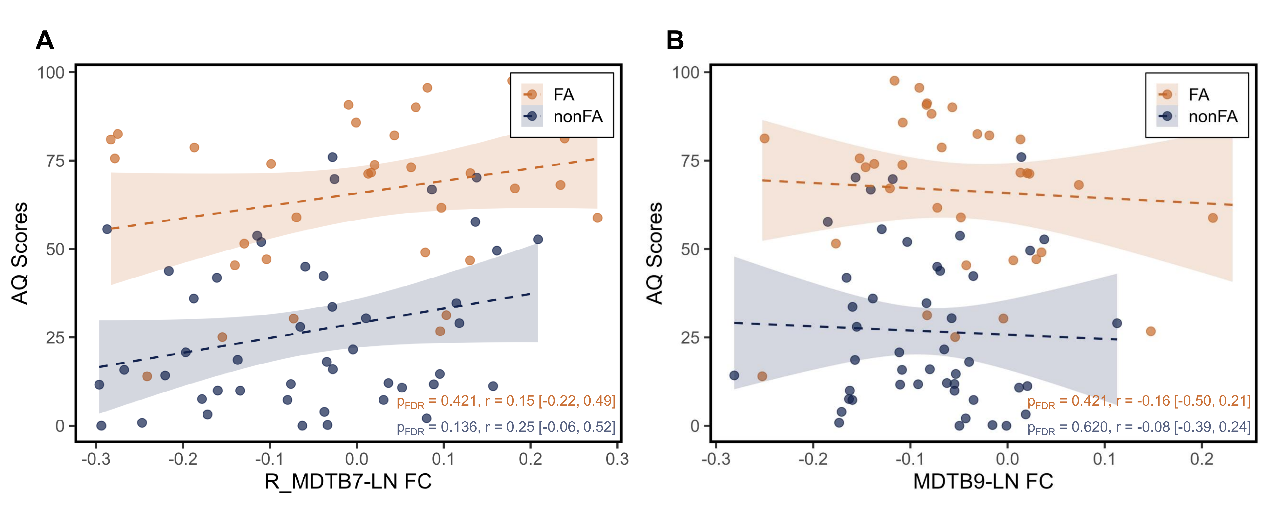


**Supplementary Figure S2 Partial correlation analyses between functional connectivity and AQ scores.**

Partial correlation analyses illustrate the relationships between (A) R_MDTB7-LN FC and (B) MDTB9-LN FC with AQ scores, adjusted for age, sex, educational level, lesion volume, and disease duration. Solid line ± shaded 95 % confidence band depicts rank-based regression; *r* and FDR-corrected *p*-values are annotated; Bold font indicates statistical significance (*p_FDR_* < 0.05).

Abbreviations: AQ = Aphasia Quotient; MDTB = Multi-Domain Task Battery; LN = classical language network; FC = functional connectivity; FA = fluent aphasia group; nonFA = non-fluent aphasia group.


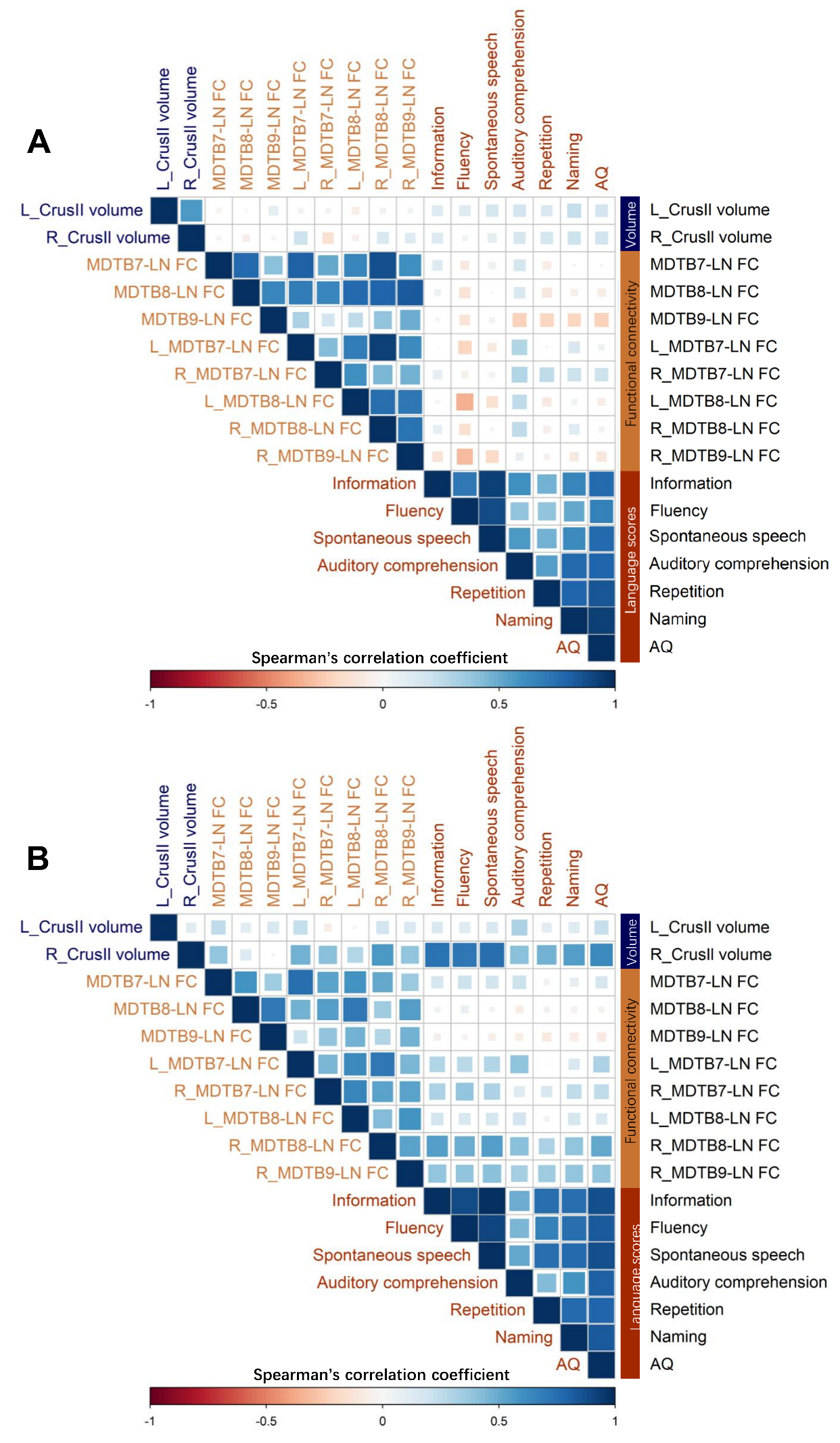


**Supplementary Figure S3 Correlations between cerebellar indicators and language performance in FA and nonFA groups.**

Correlation matrixes for (A) FA and (B) nonFA. The color bar represents the Spearman’s correlation coefficient (*r*). The size and color intensity of each square indicate the strength of the association. Variables are clustered into three categories: regional grey matter volume (dark blue), functional connectivity (orange), and language sub-scores (red).

Abbreviations: AQ = Aphasia Quotient; MDTB = Multi-Domain Task Battery; LN = classical language network; FC = functional connectivity; Crus II = cerebellar hemispheric lobule Crus II; FA = fluent aphasia group; nonFA = non-fluent aphasia group.


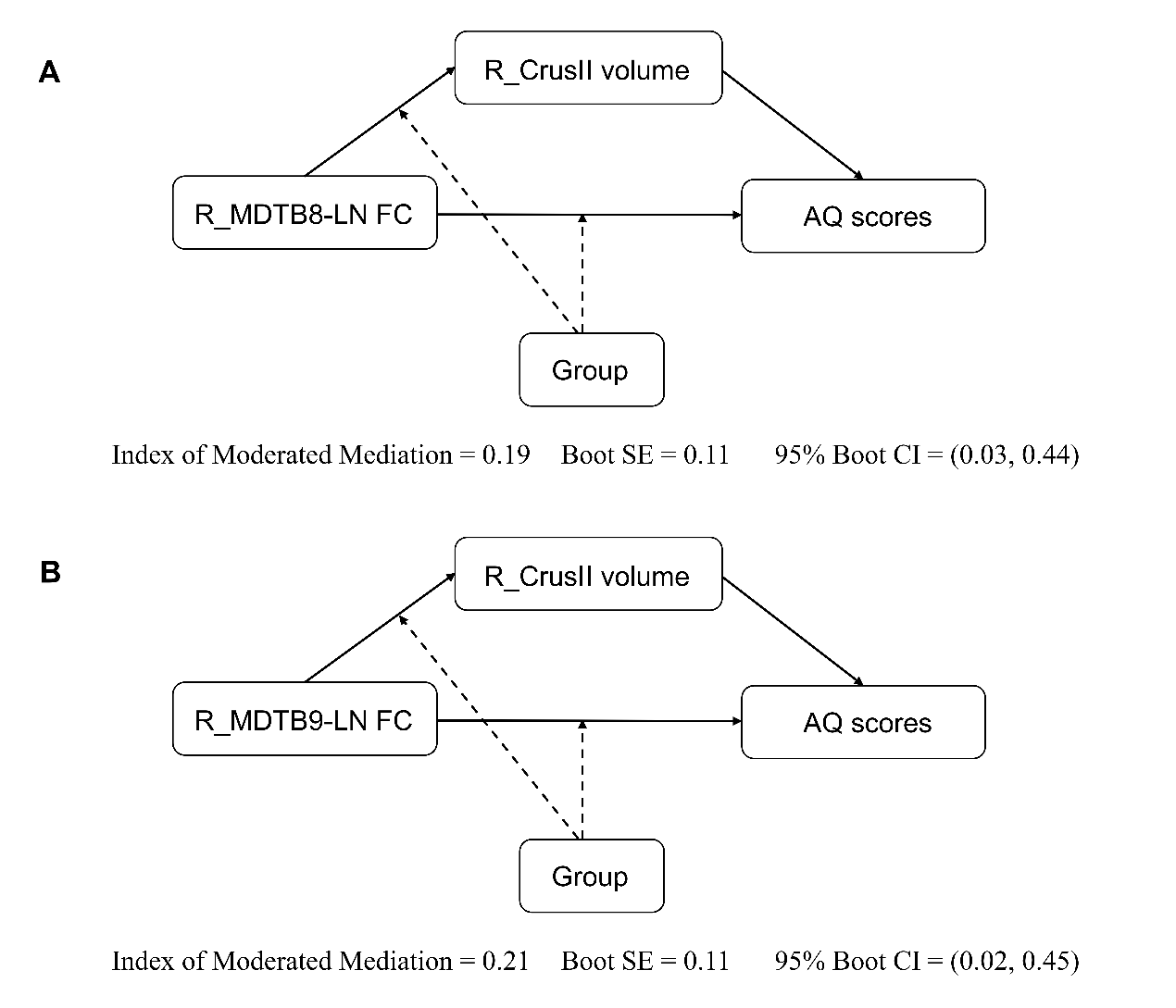


**Supplementary Figure S4 Moderated mediation analysis exploring the pathways between R_MDTB8/9-LN FC, R_Crus II volume, and AQ scores.**

R_Crus II volume as a mediator between cerebellar FC and AQ for (A) R_MDTB8-LN FC and (B) R_MDTB9-LN FC and moderated by Group. Solid lines indicate direct paths, while dashed lines represent the moderation effects of “Group” on the relationship between FC, volume, and language scores. The index of moderated mediation, Boot SE, and 95% CI are reported below each model. The 95% Boot CI that does not include zero indicates a statistically significant moderated mediation effect.

Abbreviations: Boot SE = bootstrap standard error; CI = confidence intervals; AQ = Aphasia Quotient; MDTB = Multi-Domain Task Battery; LN = classical language network; FC = functional connectivity; Crus II = cerebellar hemispheric lobule Crus II; FA = fluent aphasia group; nonFA = non-fluent aphasia group.


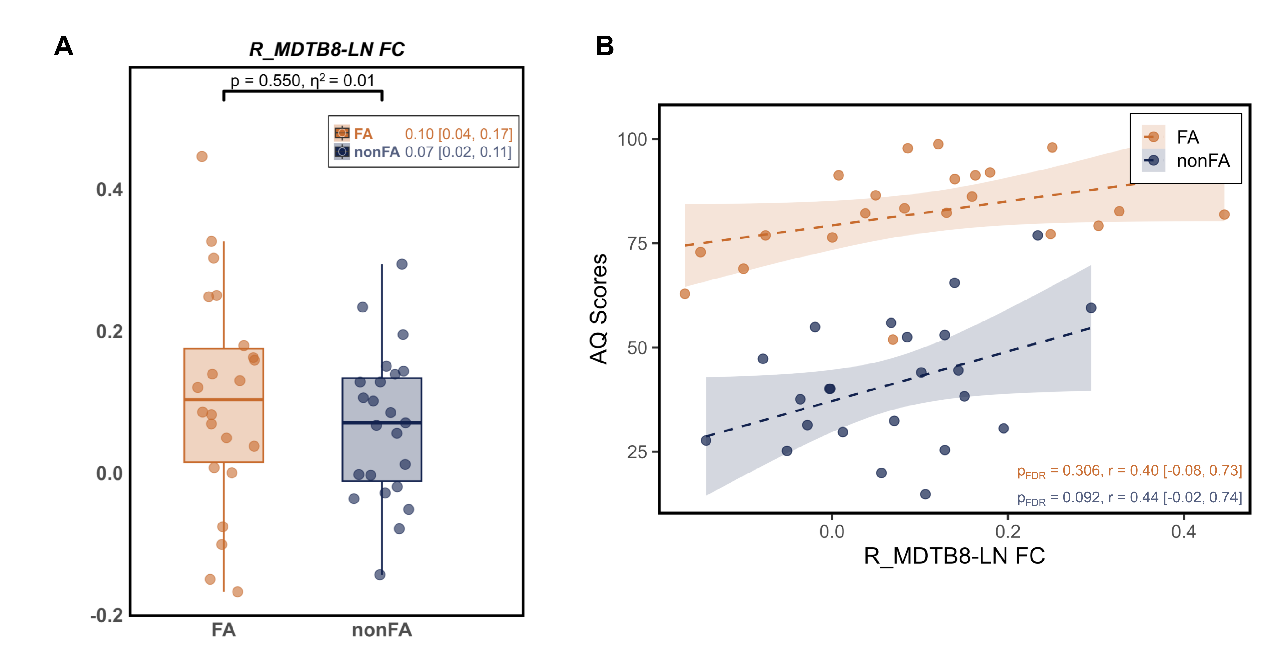


**Supplementary Figure S5 Group comparison of R_MDTB8-LN FC and its language correlate in ARC.**

(A) Group comparison of R_MDTB8-LN FC (*F_(1,39)_* = 0.36, *p* = 0.550, *η^2^* = 0.01) between FA and nonFA, adjusted for age, sex, lesion volume and disease duration. (B) Correlation between R_MDTB8-LN FC and AQ. Solid line ± shaded 95 % confidence band depicts rank-based regression; *r* and FDR-corrected *p*-values are annotated; Bold font indicates statistical significance (*p_FDR_* < 0.05).

Abbreviation: ANCOVA = Analysis of covariance; AQ = Aphasia Quotient; MDTB = Multi-Domain Task Battery; LN = classical language network; FC = functional connectivity; Crus II = cerebellar hemispheric lobule Crus II; FA = fluent aphasia group; nonFA = non-fluent aphasia group.
